# Conserved Units of Co-Expression in Bacterial Genomes: An Evolutionary Insight into Transcriptional Regulation

**DOI:** 10.1371/journal.pone.0155740

**Published:** 2016-05-19

**Authors:** Ivan Junier, Olivier Rivoire

**Affiliations:** 1 CNRS, TIMC-IMAG, F-38000 Grenoble, France; 2 Univ. Grenoble Alpes, TIMC-IMAG, F-38000 Grenoble, France; 3 CNRS, LIPhy, F-38000 Grenoble, France; 4 Univ. Grenoble Alpes, LIPhy, F-38000 Grenoble, France; Wilfrid Laurier University, CANADA

## Abstract

Genome-wide measurements of transcriptional activity in bacteria indicate that the transcription of successive genes is strongly correlated beyond the scale of operons. Here, we analyze hundreds of bacterial genomes to identify supra-operonic segments of genes that are proximal in a large number of genomes. We show that these synteny segments correspond to genomic units of strong transcriptional co-expression. Structurally, the segments contain operons with specific relative orientations (co-directional or divergent) and nucleoid-associated proteins are found to bind at their boundaries. Functionally, operons inside a same segment are highly co-expressed even in the apparent absence of regulatory factors at their promoter regions. Remote operons along DNA can also be co-expressed if their corresponding segments share a transcriptional or sigma factor, without requiring these factors to bind directly to the promoters of the operons. As evidence that these results apply across the bacterial kingdom, we demonstrate them both in the Gram-negative bacterium *Escherichia coli* and in the Gram-positive bacterium *Bacillus subtilis*. The underlying process that we propose involves only RNA-polymerases and DNA: it implies that the transcription of an operon mechanically enhances the transcription of adjacent operons. In support of a primary role of this regulation by facilitated co-transcription, we show that the transcription en bloc of successive operons as a result of transcriptional read-through is strongly and specifically enhanced in synteny segments. Finally, our analysis indicates that facilitated co-transcription may be evolutionary primitive and may apply beyond bacteria.

## Introduction

Characterizing variations of gene expression genome-wide, understanding their relation to genome organization and explaining the underlying mechanisms are fundamental challenges in biology [1]. To achieve these goals, the description of the transcriptional landscape of genomes has been refined, revealing a complex architecture not only in eukaryotes [2, 3] but also in bacteria [4, 5]. Concomitantly, genome-wide analyses of co-transcription [6–10] have led to a system-level perspective of transcriptional regulation [9, 11–13].

From a mechanistic viewpoint, many regulatory processes are known to affect gene transcription [1], with the core architecture of regulatory networks generally associated with the regulation of operons by sigma factors (SFs) and transcription factors (TFs). Yet, as we review below, these three elements alone (operons, SFs and TFs) fail to account for the most prominent features of bacterial gene co-expression, both in the Gram-negative *Escherichia coli* and in the Gram-positive *Bacillus subtilis*. Additional regulatory elements, including small metabolites [14, 15], small RNAs [16, 17], transcriptional attenuators [18], global physiological effects [19, 20] and topological properties of chromosomes [21–23] are thus expected to play a role. Yet, the implications and specificities of these mechanisms are too poorly understood to yield an alternative, reliable decomposition of genomes.

Interestingly, several previous studies suggest that transcription is primarily coordinated above the scale of operons in bacteria. For instance, in *B. subtilis*, high-resolution micro-array data has revealed large supra-operonic transcriptional units, which are controlled by SFs and essential for the adaptative properties of the bacterium [9]. In *E. coli*, micro-array data obtained under a large panel of conditions [24] has also highlighted the presence of large supra-operonic domains of coordinated gene expression dedicated to specific transcriptional responses [10]. The systematic identification of such genomic units of transcriptional coordination in every bacterium and the investigation of the underlying regulatory mechanisms remain, however, problematic for at least three reasons. First, transcription is condition-dependent so that transcriptional units may differ from one condition to the other [9, 10, 13, 25]. Second, transcription is stochastic and, even under the same condition, different units may be transcribed; in particular, transcriptional termination is rarely as sharp as transcriptional initiation. Finally, transcription is not necessarily functional and not all transcriptional units are equally relevant. Assessing functional significance is in fact challenging as this notion ultimately refers to a measure of “fitness”, which is hardly accessible given our limited knowledge of the environmental conditions under which this fitness should be evaluated.

An indirect approach to identify functionally relevant transcriptional units utilises evolutionary conservation across species as a proxy for fitness. This approach relies on the principle that features shared among a large number of distinct species must be under strong selective pressures and, therefore, are functionally significant [26]. Past studies have exploited this principle to parse out the commonalities and differences of gene regulation in different species, mostly among eukaryotes [27–29]. The limited number of species for which extensive gene expression data is available has, however, precluded a precise comparison of co-expression units [30]. Here, we circumvent this difficulty by studying the evolutionary conservation of the clustering of genes along chromosomes. In bacteria, just as in eukaryotes [30–36], “synteny”, the conservation of chromosomal proximity between genes [37–39], has indeed been shown to be tightly related to co-expression properties [40, 41] and to be useful to the inference of functional associations [42–44].

From a comprehensive analysis of synteny across a thousand annotated bacterial genomes, we thus identify “synteny segments” in every annotated genome. To this end, we define synteny segments of a particular genome as groups of consecutive genes that are co-localized both in the genome and in a significant number of other, phylogenetically distant genomes. By studying the organization of these clusters in the thousand genomes and by examining their structural and regulatory properties in two of the best characterized bacteria, *E. coli* and *B. subtilis*, we demonstrate that these synteny segments reflect supra-operonic genomic units that lie at the core of the coordination of transcription. To explain our results, we propose that “facilitated co-transcription”, the transcription of a gene (or operon) induced by the transcription of the gene (or operon) located immediately upstream, sharing or not the same orientation, is at the evolutionary origin of transcriptional regulation and still constitutes today its main basis. We find evidence for this scenario in RNA-seq data in *E. coli* and high-resolution micro-array data in *B. subtilis*. Finally, we show that our hypothesis both disposes of controversies over the evolutionary origins of gene clusters in bacterial chromosomes and allows to better apprehend the striking evolutionary properties of regulatory networks. We also discuss the relevance of this scenario beyond the bacterial kingdom.

## Results

**Fig 1:**
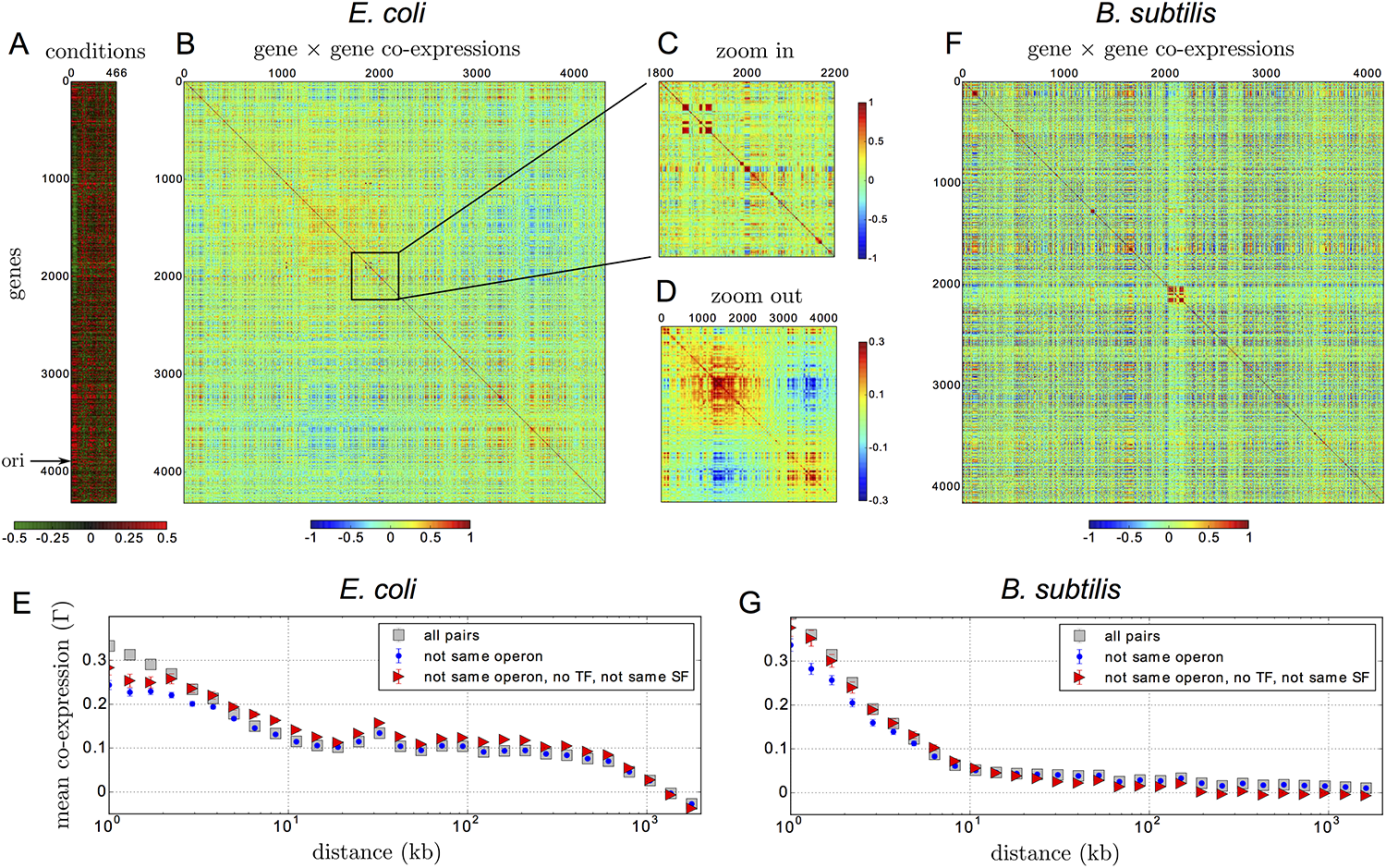
Spatial patterns of gene co-expression in *E. coli* and *B. subtilis*. **A.** For *E. coli*, we use micro-array data reporting the expression levels of 4320 genes in 466 conditions. This data is represented as a matrix with genes along rows (following their order along the chromosome) and conditions along columns: high expression appears in red and low expression in green (the data is normalized so that the mean expression of a gene across conditions is zero). **B.** The co-expression between every pair *ij* of genes is computed from their profiles of expression in the micro-array data and represented as a square matrix *C*_*ij*_ with the first gene *i* along the rows and the second *j* along the columns. The expression of two genes is positively correlated (in red) if they tend to be expressed in the same conditions and anti-correlated (in blue) if they tend to be expressed in different conditions. **C.** Zoom in the co-expression of 400 genes, showing in red small clusters of correlated genes on the scale of 10 kb (∼ 10 genes). **D.** “Zoom out” obtained by averaging the matrix on a scale of 10 kb (Gaussian filtering with a standard deviation of 10 genes), showing in red two large clusters of size ∼ 1 Mb, whose respective expressions are anti-correlated. **E.** These different features are recapitulated in the mean co-expression function Γ(*d*), defined as the average co-expression over pairs of genes at the same distance *d* along the chromosome. Γ(*d*) is computed for all pairs of genes (gray squares), for pairs of genes in distinct operons, irrespectively of their regulation by a common TF or SF (blue points), and for pairs of genes in distinct operons that are both not known to be regulated by any TF and not known to be regulated by a common SF (red triangles). **F.** Corresponding co-expression matrix for *B. subtilis*, with the presence of a highly correlated (red) cluster at the center due to the prophage SP*β* genes. **G.** For distances below ∼ 20 kb, the mean co-expression function Γ(*d*) for *B. subtilis* is similar to that in *E. coli*, with in particular a poor impact of the direct action of TFs/SFs on the enhanced co-expression observed at short distances (Γ(*d*) is computed without including the prophage SP*β* genes, which have a singular behavior).

Previous analyses have revealed similarities in the patterns of gene co-expression between *E. coli* and *B. subtilis* [45] despite their substantial evolutionary divergence (*E. coli* is a Gram-negative proteobacteria, *B. subtilis* is a Gram-positive firmicute). In this context, we first quantify the extent to which known regulatory mechanisms can explain gene co-expression in these two organisms by analyzing publicly available micro-array datasets. For *E. coli*, we use a compendium of micro-array data collected by different laboratories and normalized uniformly using a quantile normalization procedure [8]; this dataset covers 4320 genes (NC_000913 genome in NCBI reference) in 466 different conditions. For *B. subtilis*, we use the 22-base high-resolution micro-array dataset produced by the BaSysBio consortium, which covers 4162 genes (NC_000964 genome) in 104 different conditions [9]; for consistency, we first normalized this dataset using the same quantile normalization procedure as the *E. coli* dataset, even though this has no incidence on the results. For each dataset, we quantify the level of co-expression between two genes by the Pearson correlation coefficient of their transcriptomic profile (Materials and methods) and display the results in the form of a heat-map (panels B and F of Fig 1). To assess the role of operons and of TF and SF binding sites, we rely on public databases. For *E. coli*, we use the RegulonDB database [46] (last update: 02/05/2015). For *B. subtilis*, we respectively identify operons, TFs and SFs binding sites using the biocyc database (biocyc.org), the DBTBS database [47] (last update: 02/05/2015) and the comprehensive database provided by the BaSysBio consortium [9]. For the latter, we consider that an operon is directly regulated by a SF if its promoter region (up to 500 bases) contain at least one binding site of this SF (Table S2 in SOM of [9]).

### Gene co-expression is enhanced beyond the scale of operons without the involvement of TFs or SFs

In *E. coli*, our analysis reveals a hierarchical genomic organization of transcriptional co-expression in agreement with previous works (Fig 1A–1E) [45, 48]. At the bottom of this hierarchical organization, for genomic scales up to 10 kilo-bases (kb, the scale of a single gene), small clusters of positively correlated genes can be distinguished (Fig 1C). At the top of the hierarchy, we observe a global pattern of anti-correlation between two large clusters that have a genomic extension of the order of 1 Mb (1/4 of the genome length; Fig 1D). These features are recapitulated in the shape of the co-expression function Γ(*d*), defined here as the mean co-expression *C*_*ij*_ between pairs of genes separated by a given genomic distance *d*. As shown in Fig 1E (grey squares), Γ(*d*) presents a first decrease up to *d* ∼ 10 kb, which reflects the presence of the small correlated clusters. It is followed by a long plateau ending around *d* ∼ 1 Mb, which reflects the presence of the two globally anti-correlated clusters (S1 Fig). Remarkably, considering only pairs of genes in different operons in the computation of Γ(*d*) does reduce the degree of co-expression at very short scale but does not suppress its enhancement up to 10 kb (Fig 1E, blue dots).

As discussed in detail in [49], the global pattern of anti-correlation matches the genomic distribution of the main SF of *E. coli*, *σ*^70^, and correlates with the locations of the origin and terminus of replication (S1E Fig). Yet, retaining only the operons known to be transcribed with *σ*^70^, and with *σ*^70^ only, does not suppress the anti-correlations (S2A Fig). A similar conclusion is reached when considering Fis, a NAP whose activity is also associated with different phases of cell growth [23] (S1E Fig). More strikingly, considering only pairs of genes that are reported to be regulated by different SFs and not known to be regulated by any TF leaves intact the two patterns of short and long scale correlations (Fig 1E, red triangles). In fact, the majority of correlated pairs of genes does not appear to share a common TF or a common SF (S2B and S2C Fig).

As previously reported [45], at small scales *B. subtilis* has a similar pattern of co-expression (Fig 1F) with small clusters of positively correlated genes displaying enhanced co-expression at distances below *d* ∼ 10 kb (grey squares in Fig 1G). At larger scales, some major differences are apparent, including the presence of a large ∼ 200 kb cluster of strongly co-expressed genes corresponding to the expression of the subunits of a non-ribosomal peptide synthase, the PksX megacomplex. The two large anti-correlated domains found in *E. coli* are also absent, corroborating the specificity of this large-scale pattern to *γ*-proteobacteria [49]. More importantly, just as in *E. coli*, the excess of co-expression at distances below *d* ∼ 10 kb can neither be explained by the decomposition into operons (blue points in Fig 1G), nor by the direct action of TFs or SFs (red triangles).

### Beyond operons: synteny segments as units of co-expression fitting in the hierarchy of chromosomal structures

Interestingly, synteny, the conservation of gene proximity across species, can be used as a proxy of co-expression as the two properties are well known to correlate [38]. In particular, for both *E. coli* and *B. subtilis*, the more co-expressed two proximal genes are, the more likely they are to remain proximal in other distant bacterial species, independently of whether the genes belong to a same operon (S3 Fig). Following this observation, we analyzed more than 1000 complete bacterial genomes publicly available in order to identify their synteny clusters.

**Fig 2:**
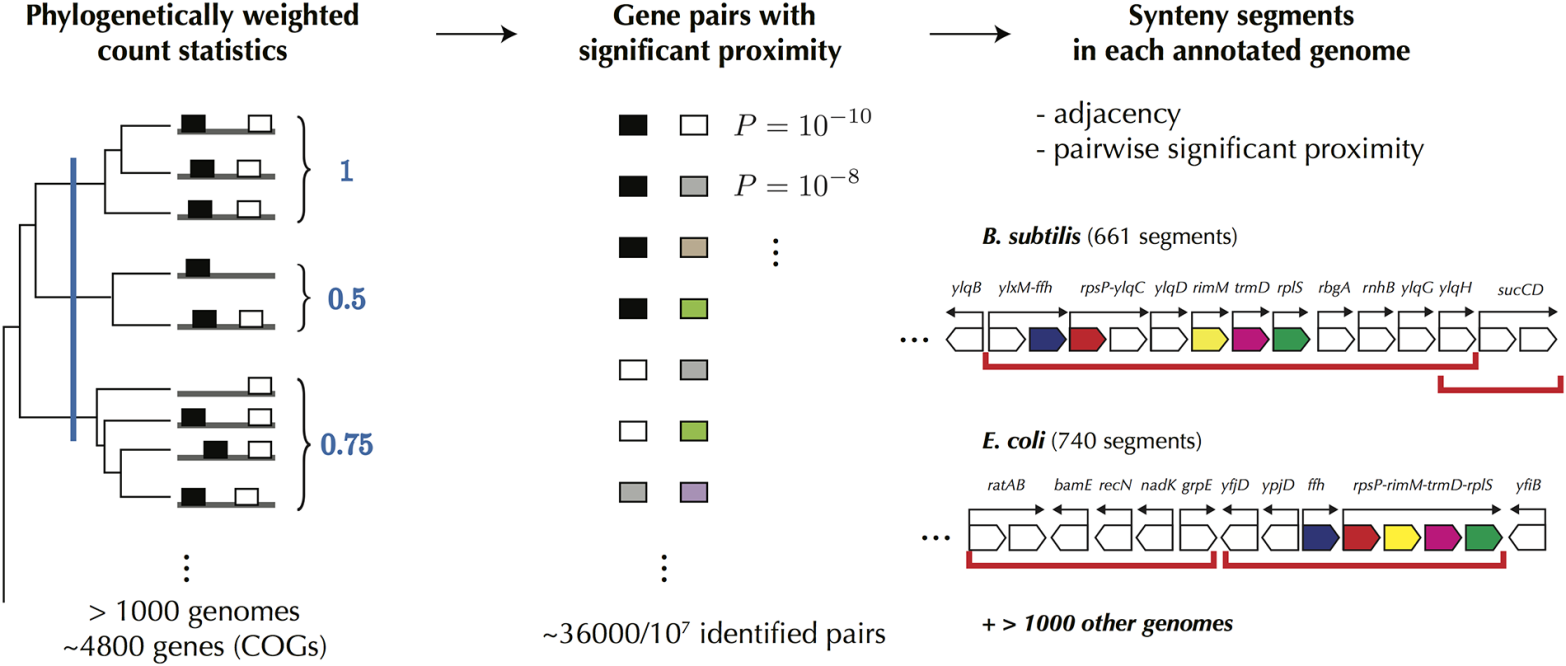
Definition of synteny segments from hundreds of genomes. Left: Synteny refers to a conservation of gene proximity along the chromosome of different species. Our analysis is based on > 1000 complete annotated genomes of bacteria. Its principle is to compute for each pair of genes the fraction of genomes in which they are proximal: if this fraction is large, the pair is considered in synteny. In practice, the calculation needs to be corrected for phylogenetic relationships between genomes since finding two genes proximal in several closely related genomes is not as meaningful as finding them in distantly related genomes (the phylogenetic depth separating close genomes from distant genomes is schematically indicated by the vertical blue line). Middle: The result is a list of pairs of genes with a p-value indicating the significance of the conservation of their proximity. We say that the pair is in synteny if the p-value is small enough, with a cut-off accounting for multiple hypothesis testing (Materials and methods). Right: In each genome, we define as synteny segment a maximal cluster of consecutive genes where every pair of genes in the cluster is in synteny. A few examples of synteny segments, delineated by red lines, are shown in *B. subtilis* and *E. coli* with white boxes representing individual genes and arrows above them indicating operons. Boxes in color indicate known orthologous genes.

We thus define the “synteny segments” of every annotated bacterial genome as the sets of genes that are both consecutive along the chromosome and proximal in a significant number of other, phylogenetically distant bacterial chromosomes (Fig 2). To build the segments, we map the genes of all genomes to one of the 4764 orthology classes defined by the Cluster of Orthologous Genes (COG) annotation [50]. We then identify the pairs of COGs that tend to remain proximal by counting the number of genomes in which two COGs are below a certain distance, itself self-consistently defined (Materials and methods) and by comparing this number to what is expected from a null model where the positions of the genes are randomly drawn according to a uniform law. To this end, we explicitly consider the effect of multiple copies of COGs and mitigate the biases coming from the uneven phylogenetic distribution of available genomes (e.g. the presence of 62 different strains of *E. coli*) by down-weighting the contributions of genomes from over-represented clades. Specifically, we follow a procedure that proved its value in other contexts [51] and weight every genome in inverse proportion to the number of genomes within a certain phylogenetic distance *δ* (left column of Fig 2). Fixing the false discovery rate to 0.005, we finally obtain, among the ∼ 10^7^ possible pairs of COGs, ∼ 36000 pairs with a significantly conserved proximity. From these pairs of genes in synteny, we define a synteny segment in a specific genome as a maximal genomic domain inside which every pair of genes is in synteny.

Taking a small false discovery rate and imposing every pair of genes in segment to be in synteny are very stringent statistical criteria to ensure that the segments that we define do reflect significant features. As a down side of taking very conservative statistical criteria, our approach is thus expected to miss not only many relevant genes in the clusters but also potentially relevant clusters entirely. This choice of minimizing type I errors (minimizing false positives) at the expense of type II errors (many false negatives) reflects our objective, which is to understand the nature of the fundamental regulatory units of bacterial genomes and not to systematically reannotate these genomes.

As a result, we find synteny segments of phylogenetically distant species that may contain similar genes but that differ in their composition (see Fig 2 for a few examples in *E. coli* and *B. subtilis*). We also observe that segments are distributed nearly uniformly along the chromosomes (S4 Fig) with a size distribution that follows, both in *E. coli* and *B. subtilis*, the size distribution of their polycistronic operons (S5 Fig). Altogether, this suggests that synteny segments represent different outcomes of a common stochastic evolutionary process [52].

**Fig 3:**
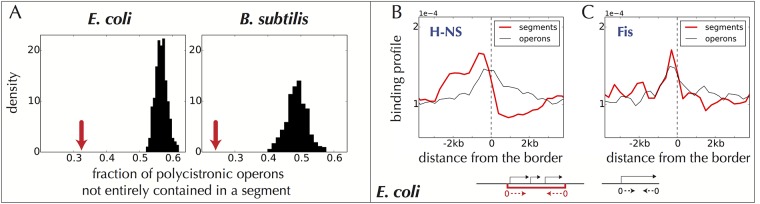
Relation of synteny segments to operons and to NAP binding sites. **A.** Operons are contained within synteny segments: few operons are only partially included inside a segment (red arrow), as compared to situations in which the synteny segments are all translated by a finite number of genes. A translation by *n* genes is defined as follows: we first label all the genes as *g*_0_, *g*_1_, *g*_2_…, following their order along the chromosome; a translation by *n* genes of a segment *g*_*k*_, *g*_*k*+1_, *g*_*k*+2_ is then *g*_*k*+*n*_, *g*_*k*+*n*+1_, *g*_*k*+*n*+2_, where the additions *k* + *n*, *k* + *n* + 1, *k* + *n* + 2 are understood modulo the total number of genes. The black histograms report a statistics over all possible translations. The distances between the red arrows and the histograms are here indicative of high significances (low p-values). **B.** In *E. coli*, the NAP H-NS shows a staircase-like binding profile at the border of synteny segments (red line) that is markably different from the binding profile around the promoters of operons not located at a border (black line, whose shape is mainly due to the TSSs). **C.** In contrast, the binding profile of the NAP Fis at the border of segments is not significantly different from that at the borders of operons. In B and C, data is from [54] and the profiles are computed with respect to the boundaries of the segments/operons as indicated by the small drawings at the bottom.

More specifically, we obtain 740 synteny segments in *E. coli* and 661 in *B. subtilis* respectively (S1 and S2 Files). These segments fit remarkably well within the known hierarchical architecture of bacterial chromosomes. At the lowest level, operons are rarely found to overlap only partially with a segment, meaning that segments contain operons (Fig 3A; see also Fig 2 for a few explicit examples). At a higher level, an analysis of the genome-wide binding profiles of various proteins onto the *E. coli* chromosome [53, 54] reveals a high preference for the nucleoid associated proteins (NAPs) Fis and H-NS to bind at the borders of synteny segments. Specifically, 359 out of 444 H-NS binding regions, and 866 out of 1246 Fis binding regions, are found within 3 kb of the border of a segment (p-values 7.10^−5^ and 5.10^−6^). In addition, we observe a strong enrichment of H-NS immediately outside synteny segments and a depletion inside them (red profile in Fig 3B); the resulting staircase-like binding profile notably differs from the binding profile around promoters of operons not located at a border (black profile). The same profile is obtained for the transcriptionally silenced extended protein occupancy domains (tsEPODs, of extension > 2 kb) identified in [53], in agreement with the fact that most of tsEPODs overlap with H-NS binding regions (S6 Fig). Fis also displays a tendency to bind immediately outside of the segments with a binding profile which, however, does not differ significantly from that of operons (Fig 3C). In contrast, the highly expressed extended protein occupancy domains (heEPODs, of extension > 2 kb, enriched in RNA polymerases) also identified in [53] are not enriched at the border of segments; instead, they tend to be located within the segments: 102 out of the 121 heEPODs overlap with the segments (p-value 4.10^−9^).

### TFs/SFs are not necessary for intra-segment co-expression but can couple the expression of distant segments

**Fig 4:**
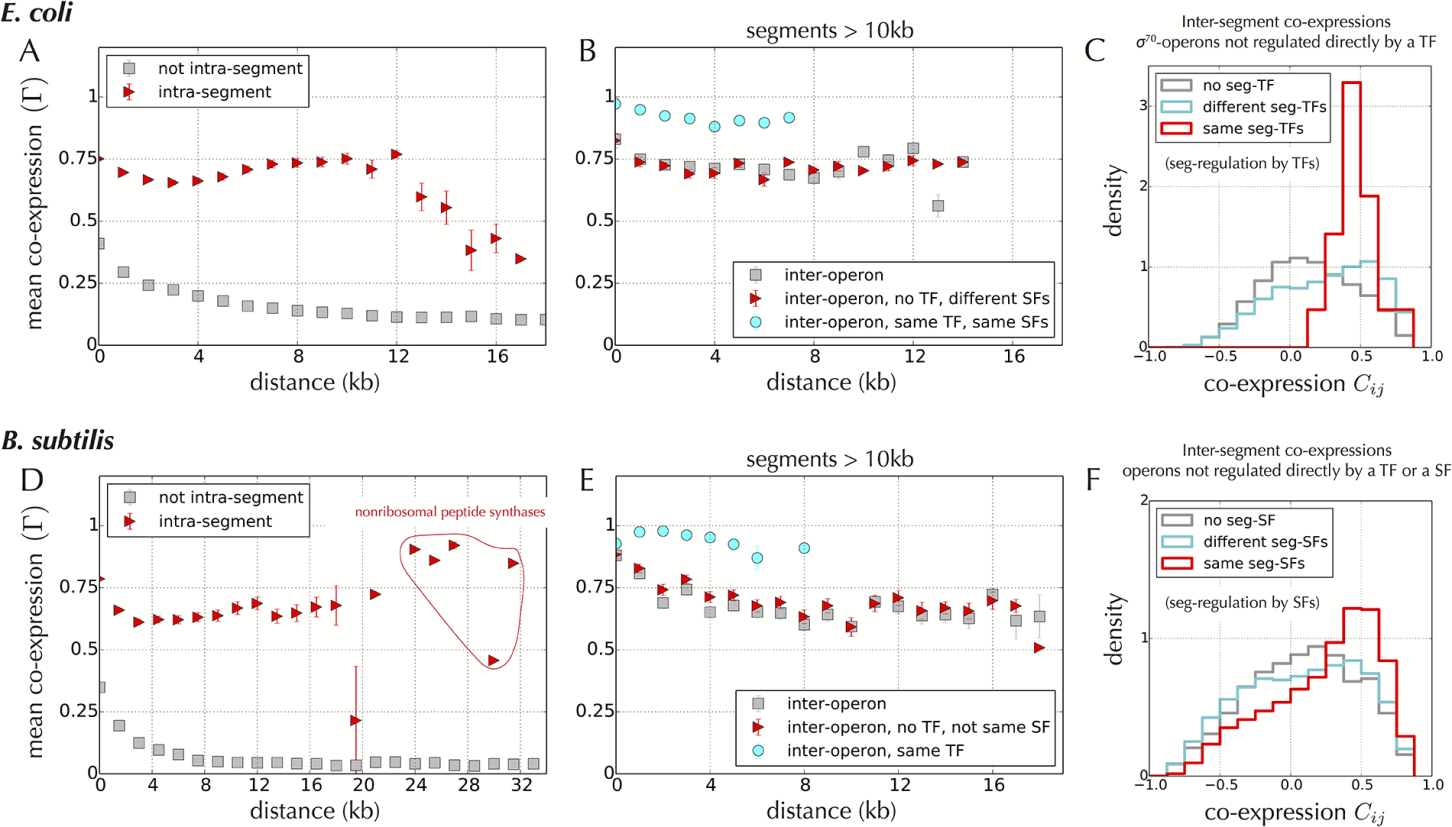
Co-expression within synteny segments in *E. coli* and *B. subtilis*. **A.** As in Fig 1E, the mean co-expression Γ(*d*) represents an average co-expression for pairs of genes at a given distance *d* along the chromosome of *E. coli*, but here it is computed for two distinct subsets of pairs, those belonging to a same segment (red triangles), and all others (gray squares). This shows that pairs of genes in a same synteny segment are significantly more co-expressed than pairs outside the segments. **B.** To discard the contribution from operons, we verify that the same results hold for pairs of genes in different operons and in segments larger than 10 kb (gray squares). The results also hold when further restricting to pairs of operons that do not share the same SF and are not regulated by any TF (red triangles), which indicates that co-regulation by TFs and SFs is not necessary to the co-expression of distinct operons inside a same segment. Considering pairs of genes regulated by the same TFs and SFs (cyan points), we observe, however, that these factors can raise the co-expression to its maximal value of 1. High levels of co-expression inside segments are observed irrespectively of the relative orientation of the operons (S8 Fig). The results also hold for small segments < 10 kb, although the average co-expression level is lower (S8 Fig). **C.** Distribution of the co-expression *C*_*ij*_ between pairs of genes that are not directly regulated by a TF and that belong to different synteny segments—only the genes regulated by the SF *σ*^70^ are considered. Gray distribution: pairs in segments without seg-TFs. Cyan distribution: pairs in segments with different sets of TFs. Red distribution: pairs in segments with exactly the same seg-TFs. The peak of the red distribution at high values of co-expression provides evidence for seg-TF regulation in *E. coli*. **D, E, F.** Essentially the same results are obtained in *B. subtilis* with one major difference: seg-regulation occurs through SFs (panel F), not TFs, in agreement with the general observation that the effect of SFs dominates over that of TFs in *B. subtilis* [9].

As respectively shown in Fig 4A and 4D for *E. coli* and *B. subtilis*, where the co-expression function Γ(*d*) of Fig 1E and 1G is compared for pairs of genes belonging to a same segment or for other pairs, co-expression occurs at high levels within synteny segments, and at low levels outside. Enhancement of co-expression within synteny segments hold equally for other phylogenetically distant bacterial species for which genome-wide transcriptional data is available in a large number of conditions (S7 Fig), corroborating the significance of synteny segments for co-expression properties in bacteria. Most notably, excluding pairs within a same operon (and segments < 10 kb, which contribute only at short distances; S8 Fig) reveals that the strong co-expression inside segments is not due to operons only, but occurs between different operons, independent of their genomic distance (Fig 4B and 4E and S7 Fig). In agreement with our analysis in Fig 1, we also find that this strong co-expression does not seem to be due to a co-regulation by TFs or SFs (red triangles in Fig 4B and 4E), although the presence of common TFs does enhance co-expression to levels close to the maximal possible value of 1 (cyan points).

Next, we find that operons that are not known to be directly regulated by a TF can be strongly co-expressed not only when they belong to the same segment but also when they belong to distant segments that share the same TF or SF signature. To demonstrate this novel layer of regulation, which we shall call “seg-regulation”, we define the “seg-TFs” (respectively, “seg-SFs”) of a segment as the set of TFs (respectively, SFs) that directly regulate at least one operon in the segment. In *E. coli*, Fig 4C shows that pairs of genes regulated by the SF *σ*^70^ but in different segments and with no TF of their own are significantly more co-expressed when they have exactly the same seg-TFs (red distribution) than when they have different seg-TFs (cyan distribution). Pairs of such genes are in fact also significantly more co-expressed when they both have a seg-TF, irrespectively of its identity, than when they both have no seg-TF, consistent with an indirect contribution from the regulation of TFs by other TFs [55]. No similar seg-TF regulation is observed in *B. subtilis*. Instead, the expression of distant segments in this bacterium appears to be coupled by SFs (Fig 4F). We find indeed that pairs of genes in different segments and with no SF of their own are significantly more co-expressed when they have exactly the same seg-SFs (red distribution) than when they have different seg-SFs (cyan distribution). This result corroborates the earlier report that most gene expression variation in *B. subtilis* is explained by changes in expression of the SFs [9].

The functional significance of seg-regulation is comforted by observing that the number of TF-regulated operons in both *E. coli* and *B. subtilis* segments is independent of the size of the segments, with on average one operon that is regulated (S9 Fig). This indeed suggests that no additional TF binding site is needed if a binding site is already present in the segment, which is therefore sufficient to regulate all operons of the segment—the same tendency is observed in *B. subtilis* for the SFs, with only a subpart of the segment that is directly regulated by a SF (S9 Fig).

Finally, let us mention a striking evolutionary link between short and long-range co-expression: pairs of genes that are distant in a genome, but in synteny in other genomes, are on average more co-expressed than those not in synteny. This phenomenon appears to be specific, in the sense that it does not apply to adjacent genes (S10 Fig). It suggests, as previously proposed in fungi [56], that operons that were previously proximal but later set apart evolve, or have evolved, similar cis-regulation.

### Operons within synteny segments are specifically organized and subject to transcriptional read-through

**Fig 5:**
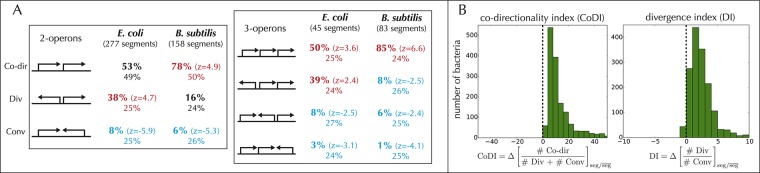
Relative orientations of operons inside synteny segments. **A.** Statistics in *E. coli* and *B. subtilis* over all synteny segments made of 2 and 3 operons exactly, showing that some organizations are over-represented (in red, with *z* > 1.65, i.e., p-value < 0.05) or under-represented (in blue, *z* < −1.65). Percentages on the first and second line correspond, respectively, to statistics within the segments and overall, for the total of 2647 operons in *E. coli* and 3450 operons in *B. subtilis*. **B.** The over-representation of co-directional genes or divergent operons inside the segments applies to every bacterial genome. This is demonstrated by computing, in each genome, indexes that reflect the statistics of gene orientations; the histograms indicate the distribution of these indexes over all genomes. For co-directionality, we define an index (CoDI) as the difference, between genes inside and outside segments, of the ratio of co-directional over divergent or convergent orientations, and observe that this index is positive in all genomes (left histogram). For divergence, we compute the difference of the ratio of divergent over convergent orientations (divergence index DI), a ratio that must be equal to 1 over an entire circular chromosome, and observe that DI is positive in almost every genome (right histogram).

To identify the mechanisms behind the strong co-expression of operons in a same synteny segment, we compare the relative orientations of operons inside and outside the segments. Inside the synteny segments made of two operons of *E. coli* and *B. subtilis*, for which operon maps have been curated for many years (Materials and methods), we first observe that convergent orientations are strongly under-represented. More specifically, in *B. subtilis* operons are mostly co-directional (in ∼ 80% of the cases), while a significant fraction of them (38% instead of the expected 25%) are divergent in *E. coli* (Fig 5A). Similarly, inside synteny segments made of three operons, patterns of co-directionality are strongly over-represented, especially in *B. subtilis*. In *E. coli*, we also find an over-representation of patterns with divergent operons.

As expected from the evolutionary conservation of synteny segments, these features are not specific to *E. coli* and *B. subtilis*, but shared across all bacterial species. To demonstrate this universal behavior, we circumvent the difficulty of defining operons by comparing the relative number of co-directional, divergent and convergent adjacent genes along each genome (Fig 5B). We observe that the ratio of co-directional over divergent or convergent orientations is systematically larger for successive genes that lie inside the synteny segments. Similarly, the ratio of divergent over convergent gene orientations, which must be 1 over an entire circular chromosome, is larger inside than outside synteny segments for almost all bacterial genomes; the genomes that do not share this property consist, without exception, of > 90% co-directional gene pairs.

**Fig 6:**
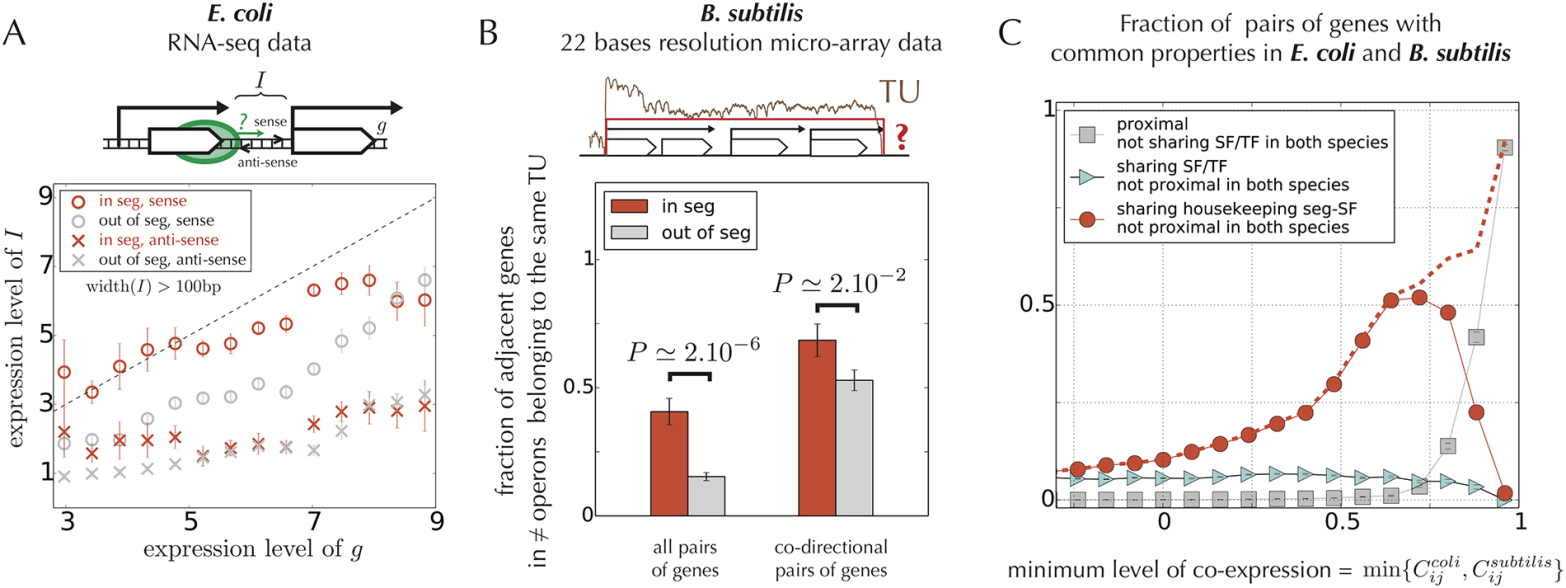
Evidence for the occurrence and evolutionary conservation of facilitated co-transcription. **A.** Evidence from RNA-seq data that transcriptional read-through is wide-spread inside the synteny segments of *E. coli*. For pairs of consecutive co-directional operons, we measure the correlation between the transcription of the first gene *g* in the downstream operon and the sense or antisense transcription of the upstream inter-operonic sequence *I* (small drawing on top). The graph shows that the sense transcription of *I* (circles) correlates strongly with the transcription of *g*, much more than anti-sense transcription (crosses), and that this correlation is stronger for operons inside a same segment (in red), except at very high transcription levels (> 8), in which case we observe a high correlation in any case. The analysis is here restricted to inter-operonic regions longer than 100 bp to exclude possibly mis-annotated operons [24]. **B.** Evidence from high-resolution micro-array data that transcriptional read-through is wide-spread inside the synteny segments of *B. subtilis*. The fraction of adjacent genes that belong to a same transcriptional unit (TU) as identified experimentally in *B. subtilis* [9] is indeed significantly higher for genes in synteny segments (red bars) than for other genes (gray bars). This is consistent with a broad overlap between the TUs and synteny segments. The two bars on the left are based on all pairs of genes in different operons and those on the right on pairs of co-directional genes in different operons. **C.** Analysis of regulatory mechanisms involved in pairs of genes with high co-expression levels in both *E. coli* and *B. subtilis* (conserved co-expression). The fraction of pairs sharing the same regulatory properties in both bacteria is represented as a function of the minimum of the co-expression levels between *B. subtilis* and *E. coli* (x-axis). We analyze three different properties: proximity (distance *d* < 20 kb), shared direct regulation by TFs or SFs, and shared seg-regulation by seg-TFs or seg-SFs (without imposing the TFs in *E. coli* and *B. subtilis* to be orthologous). We observe that the conservation of high co-expression (> 0.75) mostly results from the conservation of gene proximity (gray squares) and not from the conservation of a direct co-regulation by TFs or SFs (cyan triangles). Below 0.75, the contribution of proximity vanishes. Instead, we observe a strong relationship between the level of conserved co-expression and the tendency for being seg-regulated by the housekeeping SF (red points). The dashed red curve indicates the fraction of pairs that are explained either by proximity or by this seg-regulation, which covers the majority of pairs for co-expression levels above ∼ 0.6.

Is this singular organization of genes and operons inside synteny segments related to transcriptional co-expression? In *E. coli*, using strand-specific RNA expression profiles obtained by RNA-seq [24], we observe, indeed, that co-directional operons in a same segment are likely to be transcribed as a single large transcriptional unit. Specifically, for consecutive co-directional operons in synteny segments, we observe a correlation between the transcription of the first cistron in the downstream operon and the transcription of the upstream, non-coding inter-operonic sequence on the same strand, indicating that the gene is transcribed as a consequence of the RNA polymerase further proceeding after the transcription of the upstream operon (Fig 6A). As a control, the same analysis with the inter-operonic sequence on the opposite strand (anti-sense transcription) does not show this effect. To avoid false negatives in the identification of operons, we limited this analysis to pairs of genes separated by more than 100 bp, which is the maximal inter-gene distance considered in most operon predictions [24]. In the case of divergent operons, a previous study showed that adjacent bidirectionally transcribed genes tend to be functionally associated, with in *E. coli* many cases where one gene encodes a regulator that both controls the divergently transcribed operon and its own synthesis [43].

In *B. subtilis*, a comparison of our synteny segments to the transcriptional units (TUs) identified by the BaSysBio consortium, which also often extend beyond known operons [9], leads to the same conclusion. Fig 6B indeed shows that successive operons are significantly more likely to belong to a same TU if they belong to the same segment. This result is not explained by an enrichment in co-directional operons in both sets since it is also observed when restricting to pairs of co-directional operons (as in *E. coli*, our analysis is limited to pairs of genes separated by more than 100 bp). Two types of TUs were in fact defined in [9]: “short TUs”, which are minimal TUs found in most conditions, and “long TUs”, which are maximal TUs found in at least one condition. Here, we find a more significant overlap with the long TUs (S11 Fig), in agreement with the fact that small TUs correspond more often to single operons and therefore are not accounted for in our analysis of successive operons.

Altogether, these results indicate that transcriptional read-through, the ability of RNA polymerases to override termination signals [18] and, hence, to transcribe multiple consecutive co-directional operons into the same mRNA, is ubiquitous in synteny segments and more limited outside segments. This phenomenon thus provides a simple mechanism for seg-regulation by TFs and SFs: if an operon without any binding site near its promoter is preceded by a co-directional operon with such a site, it can be regulated by the TF or SF. There is in fact a general association between TFs and the orientations of regulated operons in *E. coli* [57]: among operons of *E. coli* preceded by a co-directional operon, only 307 are regulated by a TF while 992 are not, a difference that is highly significant (*P* ≃ 10^−7^, binomial test). The situation is similar in *B. subtilis* for the SFs: among operons preceded by a co-directional operon, only 1090 out of 2234 are regulated by a SF (*P* ≃ 10^−160^), which is in agreement with the crucial role of SFs in the co-expression patterns of this bacterium [9] (see above). Given that operons in segments are preferentially orientated co-directionally or divergently (Fig 5), altogether this suggests that synteny segments represent supra-operonic co-expression units that are controlled only by a subset of “entry points” for RNA polymerases.

### Conservation of co-expression is explained by proximity, not by the action of TFs or SFs

In contrast to relations of proximity, TFs are known to be poorly conserved [58], suggesting that pairs of genes that are co-regulated in a given species by a set of TFs may not be regulated by the same TFs, or by any TF, in a different species. The situation is somehow intermediate in the case of SFs with, on the one hand, the presence of highly conserved housekeeping SFs (*σ*^70^ in *E. coli* and SigA in *B. subtilis*) and, on the other hand, a rich diversity of stress-related SFs [59]. These considerations raise the question of the mechanisms behind the conservation of transcriptional co-expression in bacteria.

To address this problem, we consider pairs of genes that are highly co-expressed both in *E. coli* and in *B. subtilis*. We examine whether in both species these pairs of genes (i) are proximal, (ii) are directly regulated by a common TF or a SF, or (iii) share a common seg-TF or seg-SF (shared seg-regulation). To this end, we measure the fraction of pairs having one of these properties alone and report this fraction as a function of the minimum of their co-expression level in *E. coli* and in *B. subtilis* (x-axis in Fig 6C)—a large minimum co-expression indicates that co-expression is high in the two bacteria.

Our analysis reveals that strongly co-expressed pairs of genes in the two strains (*C*_*ij*_ > 0.75) are mostly proximal in the two genomes, independent of whether the genes are co-regulated by a common TF or SF (gray squares in Fig 6C). In contrast, these pairs are not enriched in non-proximal genes regulated by a common SF or by a common TF, even without requiring the TFs to be orthologous between *E. coli* and *B. subtilis* (cyan triangles in Fig 6C). More strikingly, for lower, yet significantly conserved co-expression levels (e.g. *C*_*ij*_ ∼ 0.5), we do not observe any contribution from these two mechanisms (proximity and direct co-regulation), but a strong relation to the tendency for being seg-regulated by housekeeping SFs (red points; see S12 Fig for further details). In particular, we observe that the majority of pairs of genes with conserved co-expression levels above ∼ 0.6 are either proximal or seg-regulated by these housekeeping SFs (red dashed curve).

## Discussion

Following the principle that evolutionary conservation across distant species reflects functionally important biological processes, we identified from a comparison of > 1000 bacterial genomes supra-operonic genomic units of co-expression that we call “synteny segments”. These synteny segments are consistent with previously proposed concepts of uber-operons [38], superoperons [40], persistent genes [41], clusters of pathway-related operons [60] and cluster of statistically correlated genes [61]. Structurally, they contain the operons (Fig 3A) and, in *E. coli*, the nucleoid-associated protein H-NS binds at their border (Fig 3B). The operons within segments are most often oriented co-directionally or divergently (Fig 5). Functionally, distinct operons within a same segment are strongly co-expressed, irrespectively of the presence of common transcription factors (TFs) or sigma factors (SFs).

### Facilitated co-transcription as a basic mode of regulation

Given the particular orientations of operons inside segments and the peripheral roles that TFs and SFs play in the coordination of their transcription, a parsimonious hypothesis, which we call “facilitated co-transcription”, is sufficient to summarize our findings: in absence of additional molecular factors or specific inter-gene sequence motifs, the transcription of a gene is facilitated by the transcription of the gene located immediately upstream. Under this hypothesis, the transcription of a gene facilitates the transcription of co-directional downstream genes and of divergent upstream genes.

Facilitated co-transcription may have different origins, depending on the relative orientation of the genes. For co-directional genes, a likely mechanism is transcriptional read-through, the overriding of termination signals by RNA polymerases [18], which is known to be a major source of transcripts in bacteria [62]. This conclusion is supported by our analyses of RNA-seq data in *E. coli* [24] and high-resolution micro-arrays in *B. subtilis* [9] (Fig 6A and 6B), where transcriptional read-through is found to be enhanced in synteny segments. Additional evidence for the frequent co-transcription of several successive operons is also found in a recent comprehensive analysis of transcriptional data in *M. pneumoniae* [25].

For divergent genes, the most likely physical mechanism is supercoiling [22, 23], which again does not require any factor beyond RNA polymerases and DNA: transcribing RNA polymerases generate torsional constraints that can affect the structural properties of nearby promoters [63, 64]. Bidirectional promoters are indeed known to be associated with pervasive antisense transcription [65, 66]. More generally, supercoiling is thought to affect the structuring of chromosomes over a broad range of length scales, from 10 kb to a few hundreds kb [67, 68] and is recognized as an important factor of genome-wide coordination of gene expression [21–23, 69]. In particular, the chromosome of *E. coli* has been shown to be organized into ∼ 10 kb-long independent domains of supercoiled DNA [67], the typical length scale of the co-expression clusters (Fig 1). The extended regions of DNA bound by H-NS have also been proposed to isolate these supercoiled domains from each other [53].

The co-transcription of several operons within a segment may thus not require any specific molecular machinery beyond RNA polymerases and DNA. Our hypothesis of facilitated co-transcription also implies the “seg-regulation” reported in Fig 4C and 4F: operons that are not co-regulated by TFs or SFs can nevertheless be co-expressed if their respective segments are coupled. More generally, the concept of seg-regulation provides a simple basis for understanding some of the long-range co-regulation that occurs between distant operons.

### Facilitated co-transcription as an evolutionarily primitive mode of regulation

From an evolutionary standpoint, facilitated co-transcription may represent the most primitive form of gene regulation. In this scenario, gene clustering would have come first and TF- or SF-specific regulations would represent subsequent additions, tailored to the need of each specific lineage. In support for this view, we note that TFs and their network evolve quickly compared to other genetic networks [58, 70–72], while the clustering of genes may be highly conserved throughout evolution [38]. In *E. coli*, the rewiring of gene regulatory networks has been shown to have only a marginal impact both on the genome-wide transcription and on fitness of the bacterium [73], although the biophysics of transcriptional regulation is known to impose constraints on the organization of bacterial genomes [74, 75]. Along the same line, while transcription is known to be regulated at a global level by supercoiling [76], with a demonstrated influence on fitness [77, 78], deleting Fis, one of the NAPs with which H-NS controls supercoiling, has only marginal effects, depending on the conditions under which the bacterium grows [78]. NAPs may thus also only modulate the more fundamental patterns of co-expression imposed by the relationships of proximities between genes.

As evidence for the evolutionary prevalence of regulation by facilitated co-transcription, we showed that pairs of genes with conserved strong co-expression in distant species correspond typically to genes that remained proximal or that belong to segments that share housekeeping SFs, not to genes that are directly controlled by common TFs or common SFs (Fig 6C). Together with the observation that distant segments can be further coupled by specific TFs in *E. coli* (Fig 4C) or by alternative SFs in *B. subtilis*, i.e., SFs other than the housekeeping SFs (Fig 4F and S13 Fig), this strongly suggests that the division of task between TFs and alternative SFs is evolutionarily more recent than the co-regulation of adjacent genes by facilitated co-transcription.

Our hypothesis that facilitated co-transcription of co-directional and divergent genes is at the evolutionary origin of gene clustering also disposes of the paradoxes usually associated with the evolution of operons. Although controversial [79–81], the selfish operon scenario has indeed challenged the commonly-held assumption that selection for co-regulation drove the evolution of operons [82]. In particular, it has questioned the selective advantage of evolutionary intermediates when forming a new operon by bringing together several genes and an operator. Under our hypothesis, the clustering of transcriptionally independent genes may enhance their co-expression, independent of the presence of operators. This may confer an adaptive benefit to a bacterium even before an operon is formed. Consistent with this scenario, gene clustering, just as gene gain [83], appears to be under positive selection [52]. Co-expression may, however, not be the main selective pressure behind the clustering of genes: as proposed for eukaryotic genomes, another selective pressure may come from the need to reduce fluctuations in gene expression [84].

Finally, data from *S. cerevisiae* suggests that facilitated co-transcription may be relevant beyond bacteria. Genes that are proximal in *S. cerevisiae* and whose orthologs are in synteny in bacteria show indeed a much stronger co-expression than those that are not in synteny in bacteria (S14 Fig). Moreover, whereas micro-array data associates virtually every gene of *S. cerevisiae* with at least one TF, ChIP-seq data suggests that only a small fraction of these associations stem from direct physical interactions [85], consistent with the presence of a form of seg-regulation. Higher than expected levels of co-expression between proximal genes have thus far been attributed to chromatin remodeling [35]. Facilitated co-transcription offers an alternative explanation, without, nevertheless, excluding other contributions. Beyond *S. cerevisiae*, clusters of co-expressed genes are a common feature of eukaryotic genomes [30]. As these genomes do not contain operons and have regulatory mechanisms significantly different from those of bacteria, the presence and conservation of gene clustering support the hypothesis of generic mechanisms behind the co-transcription of proximal genes. Transcriptional read-through and divergent promoters have, in fact, also been proposed to account for the conservation of gene cluster in mammals [34], and supercoiling, one mechanism that we propose for facilitated co-transcription, is also recognized as a crucial factor for the local properties of gene regulation in eukaryotes [86].

## Conclusion

Our identification by synteny of transcriptional units beyond the usual scale of operons does not call into question the existence of well-established operons as much as it challenges the very notion of “transcriptional unit”: at different times, the same genes may indeed be co-transcribed either as short operons or as longer segments. The extent of co-transcription may depend on internal and external conditions and, given these conditions, be partly stochastic. Given their evolutionary conservation, we can conclude, however, that the larger units are as much, if not more, functionally meaningful than the smaller ones.

From an evolutionary standpoint, our hypothesis that facilitated co-transcription is both historically primitive and currently primary shifts the challenge from explaining how the expressions of adjacent genes became coupled to the challenge of explaining how they became partially uncoupled [87]. This perspective confers an important role to the regulation of termination. While this aspect of the problem is beyond the scope of the present work, we note that transcriptional termination is as regulated as initiation [18], can be strongly conserved [88], and is at the heart of the hierarchical properties of co-expression in TF-depleted bacteria [25].

## Materials & Methods

### Micro-array data and transcriptional co-expression analyses

Our analysis of co-expression in *E. coli* is based on transcription profiles generated from the M^3D^ database, which concerns the expression of 4320 genes across 466 conditions normalized altogether using a quantile normalization procedure [8]. Our analysis in *B. subtilis* is based on the dataset produced by the BaSysBio consortium using 22 bases tiling resolution micro-arrays and concerns the expression of 4162 genes in 104 different conditions (for a total of 269 different experiments given the various replicates) [9]; for consistency with *E. coli*, we quantile normalized the data. For each dataset, given a gene *i* in condition *s*, we define its expression level, or activity *a*_*si*_, as the average of the values associated with the micro-array probes overlapping with the gene—a quantity already computed in the original data of *B. subtilis*. We quantify the co-expression of a pair *i*, *j* of genes by the Pearson correlation of their activities across all conditions: $C_{ij}=\sum_{s}{\bar{a}}_{si}{\bar{a}}_{sj}/\sqrt{\left(\sum_{s}{\bar{a}}_{si}^{2}\right)\left(\sum_{s}{\bar{a}}_{sj}^{2}\right)}$ where ${\bar{a}}_{is}=a_{is}-\sum_{s}a_{is}/N_{c}$ with *N*_*c*_ the number of conditions. Patterns of co-expression are visualized by representing the matrix *C*_*ij*_ with the genes ordered as along the chromosome (Fig 1B); to visualize large-scale patterns in Fig 1D, we apply a Gaussian filter with a standard deviation of 10 genes. Finally, we quantify the distance-dependence of the correlations by defining an autocorrelation function Γ(*d*) as the average value of *C*_*ij*_ over the pairs *ij* of genes at a given distance *d* ± Δ*d*, with Δ*d* = 0.5 kb. This autocorrelation can be computed using all pairs of genes, or only the pairs satisfying a given criterion, such as belonging to different operons, or comprising no gene annotated as regulated by a TF or/and by a common SF.

### Construction of synteny segments

#### Genomes

The synteny segments are defined from a systematic comparison of the relative positions of orthologous genes across multiple genomes. We downloaded all the complete and COG-annotated bacterial genomes available in the NCBI databases as of March 2015 (ftp.ncbi.nih.gov), representing a data-set of 1445 genomes. COGs are Clusters of Orthologous Genes [50], which we use to map the genes to orthology classes. COGs are defined on the principle that any group of at least three genes from distant genomes that are more similar in sequence to each other than to any other genes from the same genomes should belong to the same COG [50]. As a result, a genome may contain one, several or no gene associated with any given COG, and a gene may be associated with one or no COG. Our analysis is based on the most recent update of this approach [89], which includes 4764 different COGs.

A synteny segment in a given genome is defined as a set of consecutive genes that are also proximal in a significant number of other genomes. To identify these segments, we first define an inter-gene distance and then a criterion to assess whether two genes are proximal in a significant number of genomes. To take into account the phylogeny of the genomes when counting, we use weights that reduce the contribution of genomes with a large number of closely phylogenetically related genomes in the data-set. Finding two genes nearby in a large number of closely related genomes can thus be less significant than finding them nearby in a smaller number of more distantly related genomes.

#### Inter-gene distances

We measure the distance between two genes in base pairs, from the mid-point of their nucleotide sequences. To account for the fact that genomes may comprise several chromosomes, which may be non-circular and of different lengths, we formally circularize linear chromosomes and normalize them to a common length of *L* = 500 kb, by setting all distances exceeding *L*/2 = 250 kb to 250 kb: if *d* is the actual distance in base pairs, we thus define a normalized distance *x* by *x* = min(1, 2*d*/*L*). The normalized distance between genes on distinct chromosomes is also set to *x* = 1. As *L* = 500 kb is by far larger than the typical extension of the synteny segments that we find, the exact value of this cut-off is not determining.

#### Genome weights for counting statistics

The number *M*_*ij*_(*x*) of genomes in which genes *i* and *j* are at normalized distance *x*_*ij*_ ≤ *x* is computed as *M*_*ij*_(*x*) = ∑_*g*_ *ω*_*g*_1(*x*_*ij*_ ≤ *x*) (we consider genomes where at least one gene is present and we set *x*_*ij*_ = 1 if one of the two genes is missing), with genome weights defined by *ω*_*g*_ = 1/|{*h*: *D*_*gh*_ < *δ*}|, where |{*h*: *D*_*gh*_ < *δ*}| denotes the number of genomes *h* at phylogenetic distance at most *δ* from *g*. Here, we fix *δ* to *δ* = 0.25, which is large enough to treat as equivalent the different strains of a same species (larger values *δ* may reveal more conserved syntenic relations [52]). This weighting procedure defines an effective number of genomes as *M*′ = ∑_*g*_ *ω*_*g*_ with here *M*′ = 500—for the pair *ij*, we define the corresponding effective number of genomes, $M_{ij}^{\prime }$, by considering only the genomes where *i* and *j* are present ($M_{ij}^{\prime }\leq M^{\prime }$). We use a simple definition of evolutionary distance based on the sequence similarity of a few representative conserved genes (quantifying the phylogenetic distance between bacterial genomes is a notoriously difficult task, given that different genes in a same genome often have different histories [90]). Specifically, we selected the 10 genes associated with the COGs 126G, 173J, 202K, 2255L, 481M, 497L, 541U, 544O, 556L, 1158K. These genes were taken from a list of genes shown to reflect phylogenetic distances between bacterial strains [91], with the additional constraint that they comprise a single copy in most of the genomes of our dataset. We aligned the amino sequences of these genes with MAFFT [92] and defined the similarity between any two genes by their fraction of common amino acids in the resulting multiple sequence alignment, excluding positions with gaps in the two genes. The evolutionary similarity *S*_*gh*_ between two strains *g* and *h* was obtained by averaging these similarities over the representative genes, taking only into account those genes present in single copy in the two strains. We then defined an evolutionary distance between strains as *D*_*gh*_ = 1 − *S*_*gh*_. We checked that this procedure yields a robust estimation of evolutionary distance by repeating the analysis with subsets of only 5 of the 10 genes and verifying that it leads to equivalent results (S14 Fig).

#### Significance of proximity

Assuming a uniform distribution of genes along a circular genome of length *L*, the probability of observing a distance less than *xL*/2 between 2 given genes is just *x*. In this null model, the number *M*_*ij*_(*x*) of genomes with normalized distance *x*_*ij*_ ≤ *x* thus follows a binomial law $B(M_{ij}^{\prime },x)$, where $M_{ij}^{\prime }$ is the effective number of genomes. The probability *π*_*ij*_(*x*) of observing *M*_*ij*_(*x*) events is therefore $\pi_{ij}\left(x\right)=I_{x}\left(M_{ij}\left(x\right),M_{ij}^{\prime }-M_{ij}\left(x\right)+1\right)$, where *I*_*x*_(*m*, *n*) is the regularized incomplete beta function. The least likely and therefore most significant normalized distance ${\hat{x}}_{ij}$ between a given pair of genes *ij*, is the one minimizing *π*_*ij*_(*x*), which defines ${\hat{x}}_{ij}$ and an associated *p*-value ${\hat{\pi }}_{ij}=\pi_{ij}\left({\hat{x}}_{ij}\right)$.

To treat pairs of COGs *ij* with multiple copies (genes), we fix a gene *g*_*i*_ in *i*, count the number *n* of genes in *j* at normalized distance less than *x* to *i*, and compute the probability of the event as *p*(*x*) = 1 − (1 − *x*)^*n*^. The analysis is then performed as for a single gene (*n* = 1) but with *π*_*g*_*i*_*j*_(*x*) now standing for *π*_*g*_*i*_*j*_(*p*(*x*)), thus defining ${\hat{\pi }}_{g_{i}j}$. We then define ${\hat{\pi }}_{i|j}$ as the most significant observation when considering successively each gene *g*_*i*_ in *i*, i.e., ${\hat{\pi }}_{i|j}=\min_{g_{i}\in i}\left\{{\hat{\pi }}_{g_{i}j}\right\}$. As different numbers of genes in *i* and *j* may imply ${\hat{\pi }}_{i|j}\neq {\hat{\pi }}_{j|i}$, we finally define a symmetrical measure of significance by ${\hat{\pi }}_{ij}=\max\left({\hat{\pi }}_{i|j},{\hat{\pi }}_{j|i}\right)$.

#### Threshold of significance

Under the null model, the distribution of $y_{ij}=-\ln{\hat{\pi }}_{ij}$ is found to have an exponential tail [52], *ψ*_0_(*y*) ∼ *e*^−*ay*^, with here an exponent *a* ≃ 3.25 (S15 Fig). Given a threshold of significance π*, we compute the fraction *σ*_s_ of significant pairs, with ${\hat{\pi }}_{ij}\leq \pi^{*}$, and estimate the fraction of false positive pairs as $\sigma_{\text{fp}}=\int_{-\ln\pi^{*}}^{\infty }\psi_{0}\left(y\right)≃{\left(\pi^{*}\right)}^{a}$. Following [93], we set a threshold of significance π* by imposing a given false discovery rate FDR = *σ*_fp_/*σ*_s_, which we take to be 0.005. This leads us to a threshold π* ≃ 4.10^−4^.

#### Synteny segments

For a given genome, we call synteny segment a maximal set of consecutive genes that are all proximal between each other in a significant number of other genomes. More formally, a synteny segment is defined as a set of consecutive genes such that any two genes *i*, *j* in the segment verify ${\hat{\pi }}_{ij}<\pi^{*}$, and where none of the two genes *k*_1_, *k*_2_ at the external border of the segment verifies ${\hat{\pi }}_{ik_{r}}<\pi^{*}$ with all genes *i* in the segment—we skip genes that are not COG-annotated. The later criterion ensures that the segments are maximal, with no larger segment containing them. Note that this definition allows for overlapping segments; as a consequence, a given gene may belong to several segments, but also to no segment at all.

### Analysis of structural properties of synteny segments

#### Inclusion of operons inside segments

To relate the synteny segments to operons, we count the fraction of operons shared between two or more segments, and compare the result with counts obtained from randomized operon maps (see the legend of Fig 3 for details).

#### Profiles of NAPs with respect to the segment and operon borders

To compute the average binding profile of each NAP (here H-NS and Fis taken separately) with respect to the synteny segments of *E. coli*, we first compute a binding profile *ρ*_*k*_(*x*) for each segment *k*. To this end, we define the two borders of every segment, $x_{1}^{\left(k\right)}<x_{2}^{\left(k\right)}$, as the positions of the TSS(s) and/or stop codon(s) located at the extremities of the segment. We then define the profiles with respect to these borders as $\rho_{k}\left(x\right)=(\delta \left(x_{1}^{\left(k\right)}+x\right)+\delta \left(x_{2}^{\left(k\right)}-x\right))/2N$, with *δ*(*x*) = 1 if position *x* is bound by the NAP and 0 otherwise and with N a normalization factor ensuring that ∑_*x*_ *ρ*_*k*_(*x*) = 1. Denoting *N*_seg_ the number of segments, an average profile is finally defined by *ρ*_seg_(*x*) = Σ*_k_* *ρk*(*x*)/*N*_seg_. For comparison, we compute for each NAP the average binding profile *ρ*_op_(*x*) of the NAP with respect to the 1649 operons that are not located at the border of a segment.

#### Directionality of operons in a segment

To analyze the relative orientations of operons inside a segment, we consider segments made of 2 operons and make a statistics of the following three configurations: co-directional (on the leading or lagging strand), divergent or convergent. To compute a p-value and a z-score (number of standard deviations) for each configuration, we use a null model where the operon map is translated by an arbitrary number of operons, while the segment map is fixed (as in Fig 3). We then compute for all possible translations the resulting distributions for the numbers of co-directional, divergent and convergent orientations in the segments, and consider these distributions to be Gaussian. We analyze similarly segments made of 3 operons, in which case 4 configurations must be considered, which are represented in Fig 5A. To analyze more generally the relative orientation of genes in segments for genomes that are not annotated in operons, we consider, for a given set S of genes, the number Dir(S) of consecutive genes in S that are divergent, the number Conv(S) of consecutive genes in S that are convergent and the number Codir(S) of consecutive genes in S that are co-directional. For S consisting of an entire circular chromosome, the ratio Dir(chrom)/Conv(chrom) must be 1. To quantify the particular orientation of genes inside segments, we define a divergence index as DI = Dir(in)/Conv(in) − Dir(out)/Conv(out), where S=in (respectively, S=out) is the set of genes inside (respectively, outside) a segment. This index is computed for every genome. We also compute for every genome a co-directionality index defined as the difference of the ratio of co-directional over divergent or convergent orientations: CoDI = Codir(in)/[Conv(in) + Dir(in)] − Codir(out)/[Conv(out) + Dir(out)].

#### Transcriptional read-through analysis

To analyze transcription in non-coding, inter-operon regions of *E. coli*, we use RNA-seq data from [24], which we retrieved in the form of.sra files. RNA reads were mapped to the genome of *E. coli* K12 MG1655 using bowtie2. The number of reads per bp was then computed as the genomic coverage of the data (using genomeCoverageBed and the flags “-d -split”), with the final expression levels equal to the log-value of the mean number of reads found in the regions of interest. We considered datasets for which more than ∼ 90% of the reads were uniquely mapped. Our results are averaged over 7 different conditions corresponding to the following GEO Accession Number: GSM1104381 (sgrS- with vector), GSM1104384 (sgrS- with sgrS+ plasmid), GSM1104387 (WT in LB +*α*MG), GSM1104401 (WT in defined medium with glycerol +*α*MG), GSM1104402 (WT in defined medium with glycerol −*α*MG), GSM1104405 (sgrS- in defined medium with glycerol +*α*MG) and GSM1104408 (sgrS- in defined medium with glycerol −*α*MG). Analyzing inter-operonic transcription also requires identifying transcription start sites (TSS). We retrieved TSS datasets from the most recent update of RegulonDB (Morett dataset [46]) and from the recent dataset of Palsson’s group [5]. We combined these two datasets into a single list of TSSs, and considered operons for which the first gene had an associated TSS in the immediate upstream inter-operonic region. For genes with several potential TSSs in the inter-operonic region, we considered the closest upstream start sites. To assess whether synteny segments display any specific inter-operon transcriptional activity between co-directional consecutive operons, we further limited biases from mis-annotations by considering only inter-operon regions of size larger than 100 bp, which corresponds in *E. coli* to 243 cases of co-directional consecutive operons (29 pairs are intra-segment pairs). Considering the 7 different RNA-seq conditions of *E. coli*, we thus analyzed 203 (29 × 7) situations inside a same segment and 1498 (214 × 7) situations outside segments.

To investigate the phenomenon of transcriptional read-through in *B. subtilis*, we analyzed the tendency of adjacent genes from different operons to belong to one of the transcriptional units identified by the BaSysBio consortium. These transcriptional units represent blocks of contiguous expression that often extend the known operons of *B. subtilis* [9].

## Supporting Information

S1 File Synteny segments in *E. coli*. (TXT)

S2 File Synteny segments in *B. subtilis*. (TXT)

S1 Text Relation between gene co-expression and growth conditions. (PDF)

S1 Fig Supplementary figure. **A.** As in Fig 1A for *E. coli*, micro-array data reporting the expression levels of 4320 genes (rows) in 466 conditions (columns) with high expression in red and low expression in green. **B.** Applying a singular value decomposition to the micro-array data yields two principal components, *V*_1_ along the genes and *U*_1_ along the conditions. The co-expression matrix of Fig 1B is shown here with, above the diagonal, the genes sorted by *V*_1_: this component classifies the genes according to their contribution to one of the two anti-correlated clusters visible in Fig 1D. **C.** Same expression data as in A, but with the conditions sorted by *U*_1_ and the genes sorted by *V*_1_, thus revealing the main pattern of variation. **D.** Distribution of the conditions along the principal component *U*_1_, with different colors for the different phases of growth at which the measurements of transcriptional activity were made, showing that *U*_1_ correlates with the growth rate. **E.** Fraction of genes controlled by *σ*^70^ (gray squares) and with a binding site for the NAP Fis (red triangles) as a function of *V*_1_, showing that genes that are transcribed in growing phases (negative values of *V*_1_) are more likely to be regulated by *σ*^70^ and bound by Fis.
(PDF)

S2 Fig Supplementary figure. **A.** Transcriptional co-expression between the 1231 genes of *E. coli* having *σ*^70^ as unique SF. Genes are reordered along the first component *V*_1_ from the SVD decomposition of the data as in S1B Fig. **B.** In *E. coli*, fraction of pairs of genes belonging to different operons that share a TF, a SF or one of the two, showing that, except at very high level of co-expression (*C*_*ij*_ > 0.85), the majority (∼ 75%) of correlated pairs of genes do not share a common TF or SF. **C.** Same analysis in *B. subtilis*.
(PDF)

S3 Fig Supplementary figure. Synteny as a proxy for high co-expression. Taken two genes within 10 kb along the chromosome of a reference genome, what is the probability that these genes have orthologs within the same distance in the chromosome of another bacterium? We obtain an answer from a statistics over > 1000 bacterial genomes (left panel). This answer depends not only on the phylogenetic divergence between the query and reference genomes, but also very strongly on the level of co-expression of the two genes in the reference genome (plots): the more co-expressed are the two genes in *E. coli* (top) or in *B. subtilis* (bottom), the more likely they are to remain proximal in the chromosome of distant bacteria. The curves in the graph represent the fraction of pairs of genes within 10 kb in the reference genome (*E. coli* or *B. subtilis*) that are also within 10 kb in another genome as a function of the phylogenetic divergence between the two genomes (this divergence is measured by sequence divergence, see Materials and methods). Different colors correspond to pairs of genes with different levels of co-expression in the reference genome: proximity between highly co-expressed pairs, in red, is thus much more conserved than between weakly co-expressed pairs, in yellow. The plain lines are based on pairs of genes that do not belong to the same operon, and the dotted lines on pairs of operonic genes: this shows that the relation between co-expression and synteny extends beyond operons.
(PDF)

S4 Fig Supplementary figure. Genomic distribution of segments in *E. coli* (top) and in *B. subtilis* (bottom): the histograms of the location of the segments along the chromosome reveal a fairly uniform distribution (bin size of 65 kb). The vertical dashed lines indicate the origin (*oriC*) and terminus (*ter*) of replication. In *B. subtilis*, the depletion close to *ter* is mainly due to a poor gene annotation in this region.
(PDF)

S5 Fig Supplementary figure. Size distributions of synteny segments (solid circles) in three phylogenetically distant bacteria and of polycistronic operons in *E. coli* and in *B. subtilis* (crosses), showing a similar exponential decrease up to ∼ 10 kb.
(PDF)

S6 Fig Supplementary figure. Binding profile of tsEPODs [53] with respect to synteny segments and operons, showing, as in the case of H-NS (Fig 2D), a strikingly high density of tsEPODs at the external boundaries of segments together with a depletion inside segments (in red). In agreement with their role in transcription silencing [94], we also observe an enrichment around the promoter region, and over the first gene for operons not at the border (in black).
(PDF)

S7 Fig Supplementary figure. Co-expression analysis for two additional bacteria: **A.** *Mycoplasma pneumoniae* (classified as close to Gram-positive) and **B.** *Dickeya dadantii* (formerly *Erwinia chrysanthemi*, Gram-negative). These two bacterial strains have very different genome lengths (they contain respectively ca. 650 and 4500 protein coding genes) and lifestyles (*M. pneumoniae* is a human parasit living in the respiratory tract, *D. dadantii* is a plant pathogen); they are also phylogenetically distant from both *E. coli* and *B. subtilis* (analyzed in Fig 4). *M. pneumoniae* is known to have a tiny repertoire of TFs and a single major SF, while the regulatory network of *D. dadantii* is mostly unknown (as is the case for most bacteria). The graphs compare co-expression inside synteny segments (red triangles) to co-expression outside segments (gray squares). In both cases, only genes belonging to different operons are considered (operon map from [25] for *M. pneumoniae* and from the ProOpDB database [95] for *D. dadantii*). Co-expression levels are computed from rRNA normalized RNA-seq data obtained in 151 different conditions for *M. pneumoniae* [25] and from rRNA normalized micro-array data obtained in 32 different conditions for *D. dadantii* [96]. Although global levels of co-expression differ between strains (see [25] for a detailed analysis of co-expression properties in *M. pneumoniae*), a systematic enhancement of co-expression is observed inside synteny segments, which is nearly independent of the distance separating the genes.
(PDF)

S8 Fig Supplementary figure. **A.** The red triangles correspond to those of Fig 4B (*E. coli*), and the gray squares and cyan points show that restricting to co-directional or divergent pairs has little incidence. **B.** Similar to A, but considering the smallest segments (< 4 kb) instead of the largest ones (> 10 kb): the overall level of correlation is lower for shorter segments.
(PDF)

S9 Fig Supplementary figure. Average number of operons directly controlled by at least one TF (upper panels) or by at least one SF (lower panels) as a function of the number of operons in the segment. Results show that both in *E. coli* (left panels) and in *B. subtilis* (right panels) there is roughly a constant number (close to 1) of operons directly regulated by a TF. In contrast, most operons are directly regulated by a SF in *E. coli* (left lower panel). In *B. subtilis*, not all operons of the segment are regulated by a SF, but at least one. The dashed lines in the lower panels indicate the bisectors *y* = *x*.
(PDF)

S10 Fig Supplementary figure. Co-expression between *E. coli* genes in different operons that are not regulated by any TF and that do not share the same SF (gray squares). Pairs in synteny, independently of whether they are proximal in the chromosome of *E. coli*, are on average more co-expressed than those not in synteny (red triangles). The phenomenon appears to be specific since replacing the first gene in these pairs by its nearest neighbor not in synteny (while keeping the second gene) systematically decreases the mean level of co-expression at all distances.
(PDF)

S11 Fig Supplementary figure. Fraction of adjacent genes that belong to a same transcriptional unit (TU) in *B. subtilis* [9]. Two types of TUs are considered as proposed in [9]: “short TUs” (left panel), which are minimal TUs found in most conditions, and “long TUs” (right panel), which are maximal TUs found in at least one condition. The fraction is computed for genes inside synteny segments (red bars) and for genes outside synteny segments (gray bars). In each panel, the two bars on the left are based on all pairs of genes in different operons and those on the right on pairs of co-directional genes in different operons.
(PDF)

S12 Fig Supplementary figure. **A.** Extension of the results of Fig 6C, showing that conserved high co-expression is mostly due to a seg-regulation by housekeeping SFs (*σ*^70^ in *E. coli* and SigA in *B. subtilis*). **B.** Contribution of the seg-regulation by housekeeping SFs in each organism. **C.** Same as in B but considering only genes that belong to synteny segments, showing a strong relationship in both bacteria between gene co-expression and seg-regulation by a housekeeping SF. In B and C, the drop at high co-expression level for *B. subtilis* may either come from a too partial annotation of SF binding sites, or from the imperfect match between our synteny segments and the actual relevant co-expression unit of *B. subtilis*.
(PDF)

S13 Fig Supplementary figure. Distribution in *B. subtilis* of the co-expression *C*_*ij*_ between pairs of genes that are not directly regulated by a TF or a SF and that belong to different synteny segments. Gray distribution: pairs in segments with different sets of SFs. Red distribution: pairs in segments that have one single seg-SF, the housekeeping SigA. Cyan distribution: pairs in segments that have exactly the same seg-SFs, excluding SigA.
(PDF)

S14 Fig Supplementary figure. Co-expression for pairs of genes in synteny (red triangles) or not (gray squares) in *S. cerevisiae*. Synteny is defined from our dataset of bacterial genomes, which does not include any yeast genome. Co-expression is computed from micro-array data retrieved from the M^3D^ database [8]. Pairs of genes in synteny in bacteria are in average more co-expressed in *S. cerevisiae* than pairs that are not in synteny in bacteria.
(PDF)

S15 Fig Supplementary figure. Robustness of the calculation of evolutionary distances. We compare two evolutionary distances that were computed using two disjoint groups of 5 genes that reflect phylogenetic distances between bacterial strains (Materials and methods). One can observe a linear relationship (in red) for almost the full range of similarities, except at very low similarities. All genome pairs formed from the 1445 genomes of our dataset are reported. The dashed black line indicates the bisector *y* = *x*.
(PDF)

S16 FigSupplementary figure.Probability density of $-\text{log}(\hat{\pi })$ for the empirical data (red triangles) obtained for an effective number of genomes *M*′ = 500. For small enough values of $-\text{log}(\hat{\pi })$, the density decays exponentially with $-\text{log}(\hat{\pi })$ (black line). The deviation from an exponential at large values (gray area) indicates the conservation of co-localization. For the null model (gray points), for which we consider the same effective number of genomes but where gene positions are randomized, the exponential decay extends to larger values of $-\text{log}(\hat{\pi })$. Here, we consider a false discovery rate FDR = 0.005, leading to a threshold π* ≃ 4.10^−4^ (vertical blue line).(PDF)

## References

[1] Browning DF, Busby SJW. The regulation of bacterial transcription initiation. Nature Reviews Microbiology. 2004;2:57–65. 10.1038/nrmicro787 15035009

[2] Carninci P, Sandelin A, Lenhard B, Katayama S, Shimokawa K, Ponjavic J, et al Genome-wide analysis of mammalian promoter architecture and evolution. Nature genetics. 2006;38(6):626–635. 10.1038/ng1789 16645617

[3] David L, Huber W, Granovskaia M, Toedling J, Palm CJ, Bofkin L, et al A high-resolution map of transcription in the yeast genome. Proceedings of the National Academy of Sciences of the United States of America. 2006;103(14):5320–5325. 10.1073/pnas.0601091103 16569694 PMC1414796

[4] Cho BK, Zengler K, Qiu Y, Park YS, Knight EM, Barrett CL, et al The transcription unit architecture of the Escherichia coli genome. Nature Biotechnology. 2009;27:1043–1049. 10.1038/nbt.1582 19881496 PMC3832199

[5] Cho BK, Kim D, Knight EM, Zengler K, Palsson BØ. Genome-scale reconstruction of the sigma factor network in Escherichia coli: topology and functional states. BMC biology. 2014;12(1):4 10.1186/1741-7007-12-4 24461193 PMC3923258

[6] Gasch AP, Spellman PT, Kao CM, Carmel-Harel O, Eisen MB, Storz G, et al Genomic expression programs in the response of yeast cells to environmental changes. Molecular biology of the cell. 2000;11(12):4241–4257. 10.1091/mbc.11.12.4241 11102521 PMC15070

[7] Lee HK, Hsu AK, Sajdak J, Qin J, Pavlidis P. Coexpression analysis of human genes across many microarray data sets. Genome research. 2004;14(6):1085–1094. 10.1101/gr.1910904 15173114 PMC419787

[8] Faith JJ, Driscoll ME, Fusaro VA, Cosgrove EJ, Hayete B, Juhn FS, et al Many Microbe Microarrays Database: uniformly normalized Affymetrix compendia with structured experimental metadata. Nucleic acids research. 2007;36(Database):D866–D870. 10.1093/nar/gkm815 17932051 PMC2238822

[9] Nicolas P, Mader U, Dervyn E, Rochat T, Leduc A, Pigeonneau N, et al Condition-dependent transcriptome reveals high-level regulatory architecture in Bacillus subtilis. Science. 2012;335:1103–1106. 10.1126/science.1206848 22383849

[10] Ma Q, Yin Y, Schell MA, Zhang H, Li G, Xu Y. Computational analyses of transcriptomic data reveal the dynamic organization of the Escherichia coli chromosome under different conditions. Nucleic acids research. 2013;41:5594–5603. 10.1093/nar/gkt261 23599001 PMC3675479

[11] Segal E, Shapira M, Regev A, Pe’er D, Botstein D, Koller D, et al Module networks: identifying regulatory modules and their condition-specific regulators from gene expression data. Nature genetics. 2003;34(2):166–176. 10.1038/ng1165 12740579

[12] Bonneau R. Learning biological networks: from modules to dynamics. Nature Chemical Biology. 2008;4(11):658–664. 10.1038/nchembio.122 18936750

[13] Brooks AN, Reiss DJ, Allard A, Wu WJ, Salvanha DM, Plaisier CL, et al A system-level model for the microbial regulatory genome. Molecular Systems Biology. 2014;10:740 10.15252/msb.20145160 25028489 PMC4299497

[14] Goelzer A, Bekkal Brikci F, Martin-Verstraete I, Noirot P, Bessières P, Aymerich S, et al Reconstruction and analysis of the genetic and metabolic regulatory networks of the central metabolism of Bacillus subtilis. BMC systems biology. 2008;2:20 10.1186/1752-0509-2-20 18302748 PMC2311275

[15] Chubukov V, Gerosa L, Kochanowski K, Sauer U. Coordination of microbial metabolism. Nature Reviews Microbiology. 2014;12(5):327–340. 10.1038/nrmicro3238 24658329

[16] Waters LS, Storz G. Regulatory RNAs in Bacteria. Cell. 2009;136:615–628. 10.1016/j.cell.2009.01.043 19239884 PMC3132550

[17] Morris KV, Mattick JS. The rise of regulatory RNA. Nature Reviews Genetics. 2014;15(6):423–437. 10.1038/nrg3722 24776770 PMC4314111

[18] Henkin TM, Yanofsky C. Regulation by transcription attenuation in bacteria: how RNA provides instructions for transcription termination/antitermination decisions. BioEssays. 2002;24:700–707. 10.1002/bies.10125 12210530

[19] Klumpp S, Zhang Z, Hwa T. Growth rate-dependent global effects on gene expression in bacteria. Cell. 2009;139:1366–1375. 10.1016/j.cell.2009.12.001 20064380 PMC2818994

[20] Berthoumieux S, de Jong H, Baptist G, Pinel C, Ranquet C, Ropers D, et al Shared control of gene expression in bacteria by transcription factors and global physiology of the cell. Molecular Systems Biology. 2013;9:1–11. 10.1038/msb.2012.70 PMC3564261 23340840

[21] Dorman CJ. DNA topology and the global control of bacterial gene expression: implications for the regulation of virulence gene expression. Microbiology. 1995;141(6):1271–1280. 10.1099/13500872-141-6-1271 7670631

[22] Hatfield GW, Benham CJ. DNA topology-mediated control of global gene expression in Escherichia coli. Annual Review of Genetics. 2002;36:175–203. 10.1146/annurev.genet.36.032902.111815 12429691

[23] Travers A, Muskhelishvili G. DNA supercoiling—a global transcriptional regulator for enterobacterial growth? Nature Reviews Microbiology. 2005;3:157–169. 10.1038/nrmicro1088 15685225

[24] McClure R, Balasubramanian D, Sun Y, Bobrovskyy M, Sumby P, Genco CA, et al Computational analysis of bacterial RNA-Seq data. Nucleic acids research. 2013;41:e140–e140. 10.1093/nar/gkt444 23716638 PMC3737546

[25] Junier I, Besray Unal E, Yus E, Llorens V, Serrano L. Insights into the mechanisms of basal coordination of transcription using a genome-reduced bacterium. Cell Systems, in press, 2016. 10.1016/j.cels.2016.04.015 PMC4920955 27237741

[26] Mering Cv. STRING: a database of predicted functional associations between proteins. Nucleic acids research. 2003;31:258–261. 10.1093/nar/gkg034 12519996 PMC165481

[27] Bergmann S, Ihmels J, Barkai N. Similarities and differences in genome-wide expression data of six organisms. PLoS biology. 2004;2(1):E9 10.1371/journal.pbio.0020009 14737187 PMC300882

[28] Oldham MC, Horvath S, Geschwind DH. Conservation and evolution of gene coexpression networks in human and chimpanzee brains. Proceedings of the National Academy of Sciences of the United States of America. 2006;103(47):17973–17978. 10.1073/pnas.0605938103 17101986 PMC1693857

[29] Tsaparas P, Mariño-Ramírez L, Bodenreider O, Koonin EV, Jordan IK. Global similarity and local divergence in human and mouse gene co-expression networks. BMC Evol Biol. 2006;6:70 10.1186/1471-2148-6-70 16968540 PMC1601971

[30] Michalak P. Coexpression, coregulation, and cofunctionality of neighboring genes in eukaryotic genomes. Genomics. 2008;91:243–248. 10.1016/j.ygeno.2007.11.002 18082363

[31] Lee JM, Sonnhammer ELL. Genomic gene clustering analysis of pathways in eukaryotes. Genome research. 2003;13(5):875–882. 10.1101/gr.737703 12695325 PMC430880

[32] Hurst LD, Pál C, Lercher MJ. The evolutionary dynamics of eukaryotic gene order. Nature Reviews Genetics. 2004;5(4):299–310. 10.1038/nrg1319 15131653

[33] Singer GAC, Lloyd AT, Huminiecki LB, Wolfe KH. Clusters of co-expressed genes in mammalian genomes are conserved by natural selection. Molecular biology and evolution. 2005;22(3):767–775. 10.1093/molbev/msi062 15574806

[34] Semon M, Duret L. Evolutionary origin and maintenance of coexpressed gene clusters in mammals. Molecular Biology and Evolution. 2006;23:1715–1723. 10.1093/molbev/msl034 16757654

[35] Batada NN, Urrutia AO, Hurst LD. Chromatin remodelling is a major source of coexpression of linked genes in yeast. Trends in genetics. 2007;23:480–484. 10.1016/j.tig.2007.08.003 17822800

[36] Engström PG, Ho Sui SJ, Drivenes O, Becker TS, Lenhard B. Genomic regulatory blocks underlie extensive microsynteny conservation in insects. Genome research. 2007;17(12):1898–1908. 10.1101/gr.6669607 17989259 PMC2099597

[37] Overbeek R, Fonstein M, D’Souza M, Pusch GD, Maltsev N. The use of gene clusters to infer functional coupling. Proceedings of the National Academy of Sciences of the United States of America. 1999;96(6):2896–2901. 10.1073/pnas.96.6.2896 10077608 PMC15866

[38] Lathe WC III, Snel B, Bork P. Gene context conservation of a higher order than operons. Trends in biochemical sciences. 2000;25:474–479. 10.1016/S0968-0004(00)01663-7 11050428

[39] Tamames J. Evolution of gene order conservation in prokaryotes. Genome biology. 2001;2:RESEARCH0020 11423009 10.1186/gb-2001-2-6-research0020 PMC33396

[40] Rogozin IB, Makarova KS, Murvai J, Czabarka E, Wolf YI, Tatusov RL, et al Connected gene neighborhoods in prokaryotic genomes. Nucleic acids research. 2002;30:2212–2223. 10.1093/nar/30.10.2212 12000841 PMC115289

[41] Fang G, Rocha EP, Danchin A. Persistence drives gene clustering in bacterial genomes. BMC genomics. 2008;9:4 10.1186/1471-2164-9-4 18179692 PMC2234087

[42] Ermolaeva MD, White O, Salzberg SL. Prediction of operons in microbial genomes. Nucleic acids research. 2001;29(5):1216–1221. 10.1093/nar/29.5.1216 11222772 PMC29727

[43] Korbel JO, Jensen LJ, Von Mering C, Bork P. Analysis of genomic context: prediction of functional associations from conserved bidirectionally transcribed gene pairs. Nature Biotechnology. 2004;22:911–917. 10.1038/nbt988 15229555

[44] Janga SC, Collado-Vides J, Moreno-Hagelsieb G. Nebulon: a system for the inference of functional relationships of gene products from the rearrangement of predicted operons. Nucleic acids research. 2005;33(8):2521–2530. 10.1093/nar/gki545 15867197 PMC1088069

[45] Carpentier AS, Torrésani B, Grossmann A, Hénaut A. Decoding the nucleoid organisation of Bacillus subtilis and Escherichia coli through gene expression data. BMC genomics. 2005;6:84 10.1186/1471-2164-6-84 15938745 PMC1177944

[46] Salgado H, Peralta-Gil M, Gama-Castro S, Santos-Zavaleta A, Muniz-Rascado L, Garcia-Sotelo JS, et al RegulonDB v8.0: omics data sets, evolutionary conservation, regulatory phrases, cross-validated gold standards and more. Nucleic acids research. 2012;41(D1):D203–D213. 10.1093/nar/gks1201 23203884 PMC3531196

[47] Sierro N, Makita Y, de Hoon M, Nakai K. DBTBS: a database of transcriptional regulation in Bacillus subtilis containing upstream intergenic conservation information. Nucleic Acids Research. 2008;36(Database issue):D93–6. 10.1093/nar/gkm910 17962296 PMC2247474

[48] Jeong KS, Ahn J, Khodursky AB. Spatial patterns of transcriptional activity in the chromosome of Escherichia coli. Genome biology. 2004;5:R86 10.1186/gb-2004-5-11-r86 15535862 PMC545777

[49] Sobetzko P, Travers A, Muskhelishvili G. Gene order and chromosome dynamics coordinate spatiotemporal gene expression during the bacterial growth cycle. Proceedings of the National Academy of Sciences of the United States of America. 2012;109:E42–E50. 10.1073/pnas.1108229109 22184251 PMC3258614

[50] Tatusov RL, Galperin MY, Natale DA, Koonin EV. The COG database: a tool for genome-scale analysis of protein functions and evolution. Nucleic acids research. 2000;28:33–36. 10.1093/nar/28.1.33 10592175 PMC102395

[51] Morcos F, Pagnani A, Lunt B, Bertolino A, Marks DS, Sander C, et al Direct-coupling analysis of residue coevolution captures native contacts across many protein families. Proceedings of the National Academy of Sciences of the United States of America. 2011;108:E1293–301. 10.1073/pnas.1111471108 22106262 PMC3241805

[52] Junier I, Rivoire O. Synteny in bacterial genomes: inference, organization and evolution; 2013.

[53] Vora T, Hottes AK, Tavazoie S. Protein occupancy landscape of a bacterial genome. Molecular Cell. 2009;35:247–253. 10.1016/j.molcel.2009.06.035 19647521 PMC2763621

[54] Kahramanoglou C, Seshasayee ASN, Prieto AI, Ibberson D, Schmidt S, Zimmermann J, et al Direct and indirect effects of H-NS and Fis on global gene expression control in Escherichia coli. Nucleic acids research. 2011;39:2073–2091. 10.1093/nar/gkq934 21097887 PMC3064808

[55] Martínez-Antonio A, Janga SC, Thieffry D. Functional organisation of Escherichia coli transcriptional regulatory network. Journal of molecular biology. 2008;381(1):238–247. 10.1016/j.jmb.2008.05.054 18599074 PMC2726282

[56] Wang GZ, Chen WH, Lercher MJ. Coexpression of linked gene pairs persists long after their separation. Genome Biology and Evolution. 2011;3:565–570. 10.1093/gbe/evr049 21737396 PMC3156566

[57] Hershberg R, Yeger-Lotem E, Margalit H. Chromosomal organization is shaped by the transcription regulatory network. Trends in genetics. 2005;21:138–142. 10.1016/j.tig.2005.01.003 15734572

[58] Babu MM, Teichmann SA, Aravind L. Evolutionary dynamics of prokaryotic transcriptional regulatory networks. Journal of Molecular Biology. 2006;358:614–633. 10.1016/j.jmb.2006.02.019 16530225

[59] Gruber TM, Gross CA. Multiple sigma subunits and the partioning of bacterial transcription space. Annual Review of Microbiology. 2003;57:441–466. 10.1146/annurev.micro.57.030502.090913 14527287

[60] Yin Y, Zhang H, Olman V, Xu Y. Genomic arrangement of bacterial operons is constrained by biological pathways encoded in the genome. Proceedings of the National Academy of Sciences of the United States of America. 2010;107:6310–6315. 10.1073/pnas.0911237107 20308592 PMC2851940

[61] Junier I, Hérisson J, Képès F. Genomic organization of evolutionarily correlated genes in bacteria: limits and strategies. Journal of Molecular Biology. 2012;419:369–386. 10.1016/j.jmb.2012.03.009 22446685

[62] Wade JT, Grainger DC. Pervasive transcription: illuminating the dark matter of bacterial transcriptomes. Nature Reviews Microbiology. 2014;12:647–653. 10.1038/nrmicro3316 25069631

[63] Liu LF, Wang JC. Supercoiling of the DNA template during transcription. Proceedings of the National Academy of Sciences of the United States of America. 1987;84:7024–7027. 10.1073/pnas.84.20.7024 2823250 PMC299221

[64] Meyer S, Beslon G. Torsion-mediated interaction between adjacent genes. PLoS Computational Biology. 2014;10:e1003785 10.1371/journal.pcbi.1003785 25188032 PMC4154641

[65] Dornenburg JE, Devita AM, Palumbo MJ, Wade JT. Widespread antisense transcription in Escherichia coli. mBio. 2010;1 10.1128/mBio.00024-10 PMC2912661 20689751

[66] Lasa I, Toledo-Arana A, Dobin A, Villanueva M, de los Mozos IR, Vergara-Irigaray M, et al Genome-wide antisense transcription drives mRNA processing in bacteria. Proceedings of the National Academy of Sciences of the United States of America. 2011;108:20172–20177. 10.1073/pnas.1113521108 22123973 PMC3250193

[67] Postow L, Hardy CD, Arsuaga J, Cozzarelli NR. Topological domain structure of the Escherichia coli chromosome. Genes & Development. 2004;18:1766–1779. 10.1101/gad.1207504 15256503 PMC478196

[68] Le TBK, Imakaev MV, Mirny LA, Laub MT. High-resolution mapping of the spatial organization of a bacterial chromosome. Science. 2013;342:731–734. 10.1126/science.1242059 24158908 PMC3927313

[69] Peter BJ, Arsuaga J, Breier AM, Khodursky AB, Brown PO, Cozzarelli NR. Genomic transcriptional response to loss of chromosomal supercoiling in Escherichia coli. Genome biology. 2004;5(11):R87 10.1186/gb-2004-5-11-r87 15535863 PMC545778

[70] Lozada-Chávez I, Janga SC, Collado-Vides J. Bacterial regulatory networks are extremely flexible in evolution. Nucleic Acids Research. 2006;34(12):3434–3445. 10.1093/nar/gkl423 16840530 PMC1524901

[71] Price MN, Dehal PS, Arkin AP. Horizontal gene transfer and the evolution of transcriptional regulation in Escherichia coli. Genome biology. 2008;9(1):R4 10.1186/gb-2008-9-1-r4 18179685 PMC2395238

[72] Shou C, Bhardwaj N, Lam HYK, Yan KK, Kim PM, Snyder M, et al Measuring the evolutionary rewiring of biological networks. PLoS Computational Biology. 2011;7:e1001050 10.1371/journal.pcbi.1001050 21253555 PMC3017101

[73] Isalan M, Lemerle C, Michalodimitrakis K, Horn C, Beltrao P, Raineri E, et al Evolvability and hierarchy in rewired bacterial gene networks. Nature. 2008;452:840–845. 10.1038/nature06847 18421347 PMC2666274

[74] Kolesov G, Wunderlich Z, Laikova ON, Gelfand MS, Mirny LA. How gene order is influenced by the biophysics of transcription regulation. Proceedings of the National Academy of Sciences. 2007;104(35):13948–13953. 10.1073/pnas.0700672104 PMC1955771 17709750

[75] Janga SC, Salgado H, Martínez-Antonio A. Transcriptional regulation shapes the organization of genes on bacterial chromosomes. Nucleic acids research. 2009; p. gkp231. 10.1093/nar/gkp231 PMC2699516 19372274

[76] Blot N, Mavathur R, Geertz M, Travers A, Muskhelishvili G. Homeostatic regulation of supercoiling sensitivity coordinates transcription of the bacterial genome. EMBO reports. 2006;7:710–715. 10.1038/sj.embor.7400729 16799466 PMC1500834

[77] Crozat E, Philippe N, Lenski RE, Geiselmann J, Schneider D. Long-term experimental evolution in Escherichia coli. XII. DNA topology as a key target of selection. Genetics. 2005;169:523–532. 10.1534/genetics.104.035717 15489515 PMC1449116

[78] Crozat E, Winkworth C, Gaffe J, Hallin PF, Riley MA, Lenski RE, et al Parallel genetic and phenotypic evolution of DNA superhelicity in experimental populations of Escherichia coli. Molecular Biology and Evolution. 2010;27:2113–2128. 10.1093/molbev/msq099 20392810

[79] Pál C, Hurst LD. Evidence against the selfish operon theory. Trends in genetics. 2004;20:232–234. 10.1016/j.tig.2004.04.001 15145575

[80] Price MN, Huang KH, Arkin AP, Alm EJ. Operon formation is driven by co-regulation and not by horizontal gene transfer. Genome Research. 2005;15:809–819. 10.1101/gr.3368805 15930492 PMC1142471

[81] Ballouz S, Francis AR, Lan R, Tanaka MM. Conditions for the evolution of gene clusters in bacterial genomes. PLoS computational biology. 2010;6(2):e1000672 10.1371/journal.pcbi.1000672 20168992 PMC2820515

[82] Lawrence JG, Roth JR. Selfish operons: horizontal transfer may drive the evolution of gene clusters. Genetics. 2002;143:1843–1860. 10.1093/genetics/143.4.1843 PMC1207444 8844169

[83] Snel B, Bork P, Huynen MA. Genomes in flux: the evolution of archaeal and proteobacterial gene content. Genome Research. 2002;12:17–25. 10.1101/gr.176501 11779827

[84] Batada NN, Hurst LD. Evolution of chromosome organization driven by selection for reduced gene expression noise. Nature genetics. 2007;39(8):945–949. 10.1038/ng2071 17660811

[85] Geistlinger L, Csaba G, Dirmeier S, Küffner R, Zimmer R. A comprehensive gene regulatory network for the diauxic shift in Saccharomyces cerevisiae. Nucleic acids research. 2013;41:8452–8463. 10.1093/nar/gkt631 23873954 PMC3794591

[86] Kouzine F, Levens D. Supercoil-driven DNA structures regulate genetic transactions. Frontiers in bioscience: a journal and virtual library. 2007;12:4409–4423. 10.2741/2398 17485385

[87] Singh SS, Singh N, Bonocora RP, Fitzgerald DM, Wade JT, Grainger DC. Widespread suppression of intragenic transcription initiation by H-NS. Genes & Development. 2014;28:214–219. 10.1101/gad.234336.113 24449106 PMC3923964

[88] Merino E, Yanofsky C. Transcription attenuation: a highly conserved regulatory strategy used by bacteria. Trends in genetics. 2005;21:260–264. 10.1016/j.tig.2005.03.002 15851059

[89] Galperin MY, Makarova KS, Wolf YI, Koonin EV. Expanded microbial genome coverage and improved protein family annotation in the COG database. Nucleic acids research. 2014; p. gku1223. 10.1093/nar/gku1223 PMC4383993 25428365

[90] Gabaldón T, Koonin EV. Functional and evolutionary implications of gene orthology. Nature Reviews Genetics. 2013;14:360–366. 10.1038/nrg3456 23552219 PMC5877793

[91] Zeigler DR. Gene sequences useful for predicting relatedness of whole genomes in bacteria. International Journal of Systematic and Evolutionary Biology. 2003;53:1893–1900. 10.1099/ijs.0.02713-0 14657120

[92] Katoh K, Kuma K, Toh H, Miyata T. MAFFT version 5: improvement in accuracy of multiple sequence alignment. Nucleic acids research. 2005;33:511–518. 10.1093/nar/gki198 15661851 PMC548345

[93] Benjamini Y, Hochberg Y. Controlling the false discovery rate: a practical and powerful approach to multiple testing. Journal of the Royal Statistical Society Series B. 1995;57:289–300.

[94] Dorman CJ. H-NS, the genome sentinel. Nature Reviews Microbiology. 2007;5(2):157–161. 10.1038/nrmicro1598 17191074

[95] Taboada B, Ciria R, Martinez-Guerrero CE, Merino E. ProOpDB: prokaryotic operon database. Nucleic acids research. 2012;40(D1):D627–D631. 10.1093/nar/gkr1020 22096236 PMC3245079

[96] Jiang X, Sobetzko P, Nasser W, Reverchon S, Muskhelishvili G. Chromosomal Stress-Response Domains Govern the Spatiotemporal Expression of the Bacterial Virulence Program. MBio. 2015;6(3):e00353–15. 10.1128/mBio.00353-15 25922390 PMC4436070

