# Supplementary material for: Conserved Units of Co-Expression in Bacterial Genomes: An Evolutionary Insight into Transcriptional Regulation
Source: PLoS One. 2016 May 19;11(5):e0155740. doi: 10.1371/journal.pone.0155740 (PMC4873041; doi:10.1371/journal.pone.0155740)
Supplement: S1 Text — (PDF) [file pone.0155740.s003.pdf]

S1 TEXT

Relation between gene co-expression and growth conditions

The two globally anti-correlated clusters seen in Fig 1D can be interpreted functionally by relating them to the conditions under which the genes are expressed. To this end, a statistical method extending principal component analysis known as singular value decomposition can be applied, which reorders the genes and the conditions in a consistent way, according to their main axes of variation [1]. Specifically, the singular value decomposition of the transcription profile matrix $\bar{a}_{si}$ is of the form $\bar{a}_{si} = \sum \rho_k u_{sk} v_{ik}$, with $\rho_1 \geq \rho_2 \geq \cdots \geq 0$ the set of singular values. $\{u_s\}_{s=1\ldots\#\text{conditions}}$ and $\{v_i\}_{i=1\ldots\#\text{genes}}$ are, respectively, orthonormal basis of the gene space and of the condition space. The top singular vectors U_1 and V_1 have components $(U_1)_s = u_{s1}$ and $(V_1)_i = v_{i1}$, and define the main axes of variation in the gene space and condition space, respectively.

As a result, we obtain an ordered list of genes with the most anti-correlated genes at the two extremes, and an ordered list of conditions depending on whether they induce one or the other set of genes (S1 Fig B-C). These lists indicate a simple interpretation of the two globally anti-correlated gene clusters in terms of phase of cell growth. Indeed, one gene cluster is preferentially expressed during exponential growth and the other during stationary phase (S1 Fig D). This association of different growth rates with different overall patterns of gene expression is well recognized [2]. The preferential location of the anti-correlated genes on different halves of the genome is consistent with previous analyses [3].

References

[1] Alter O, Brown PO, Botstein D. Singular value decomposition for genome-wide expression data processing and modeling. Proc Natl Acad Sci USA. 2000;97:10101–10106.

[2] Shoval O, Sheftel H, Shinar G, Hart Y, Ramote O, Mayo A, et al. Evolutionary trade-offs, Pareto optimality, and the geometry of phenotype space. Science. 2012;336:1157–1160.

[3] Sobetzko P, Travers A, Muskhelishvili G. Gene order and chromosome dynamics coordinate spatiotemporal gene expression during the bacterial growth cycle. Proc Natl Acad Sci USA. 2012;109:E42–E50.
