# Supplementary figures and images for: Conserved Units of Co-Expression in Bacterial Genomes: An Evolutionary Insight into Transcriptional Regulation
Source: PLoS One. 2016 May 19;11(5):e0155740. doi: 10.1371/journal.pone.0155740 (PMC4873041; doi:10.1371/journal.pone.0155740)

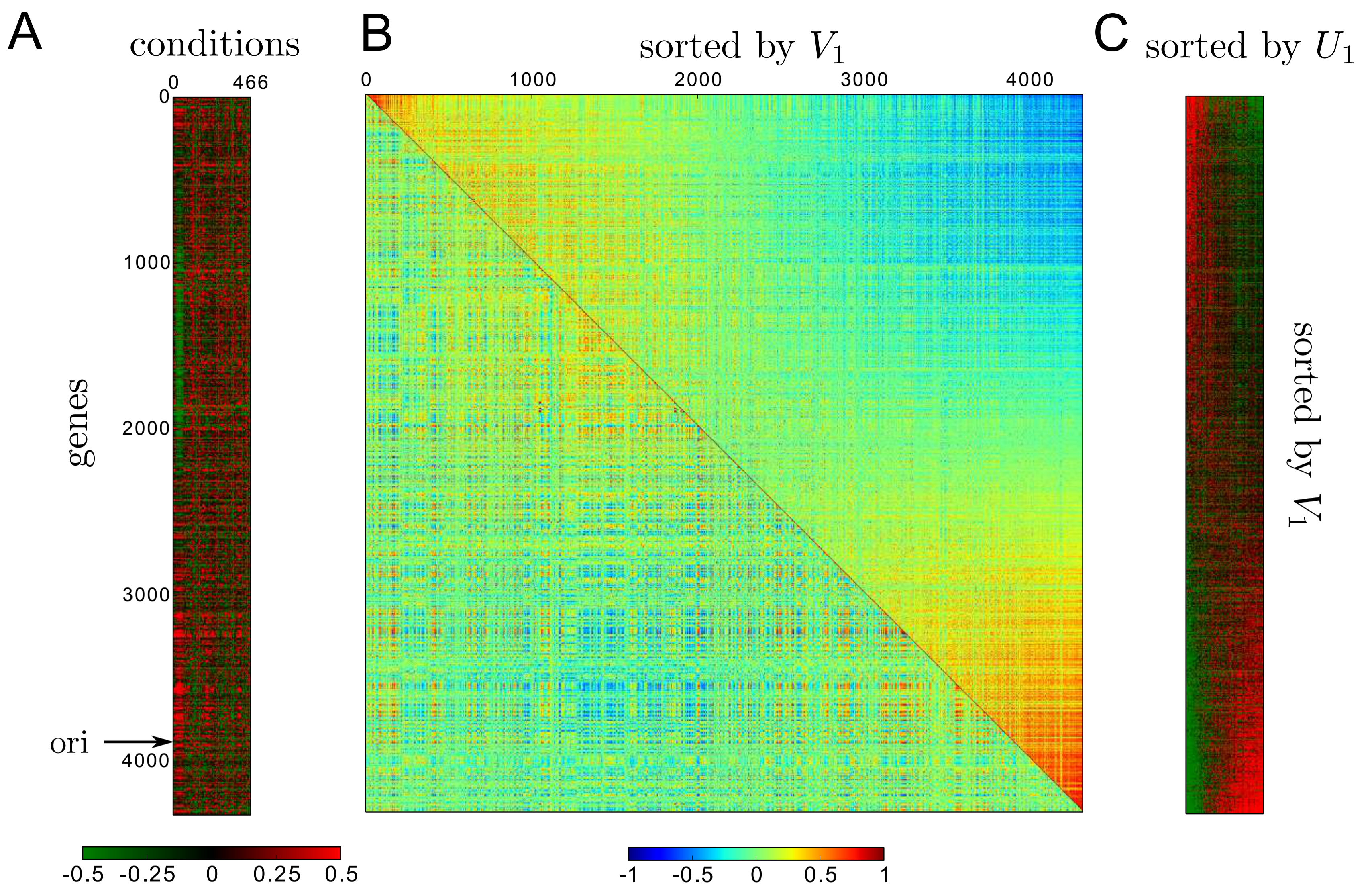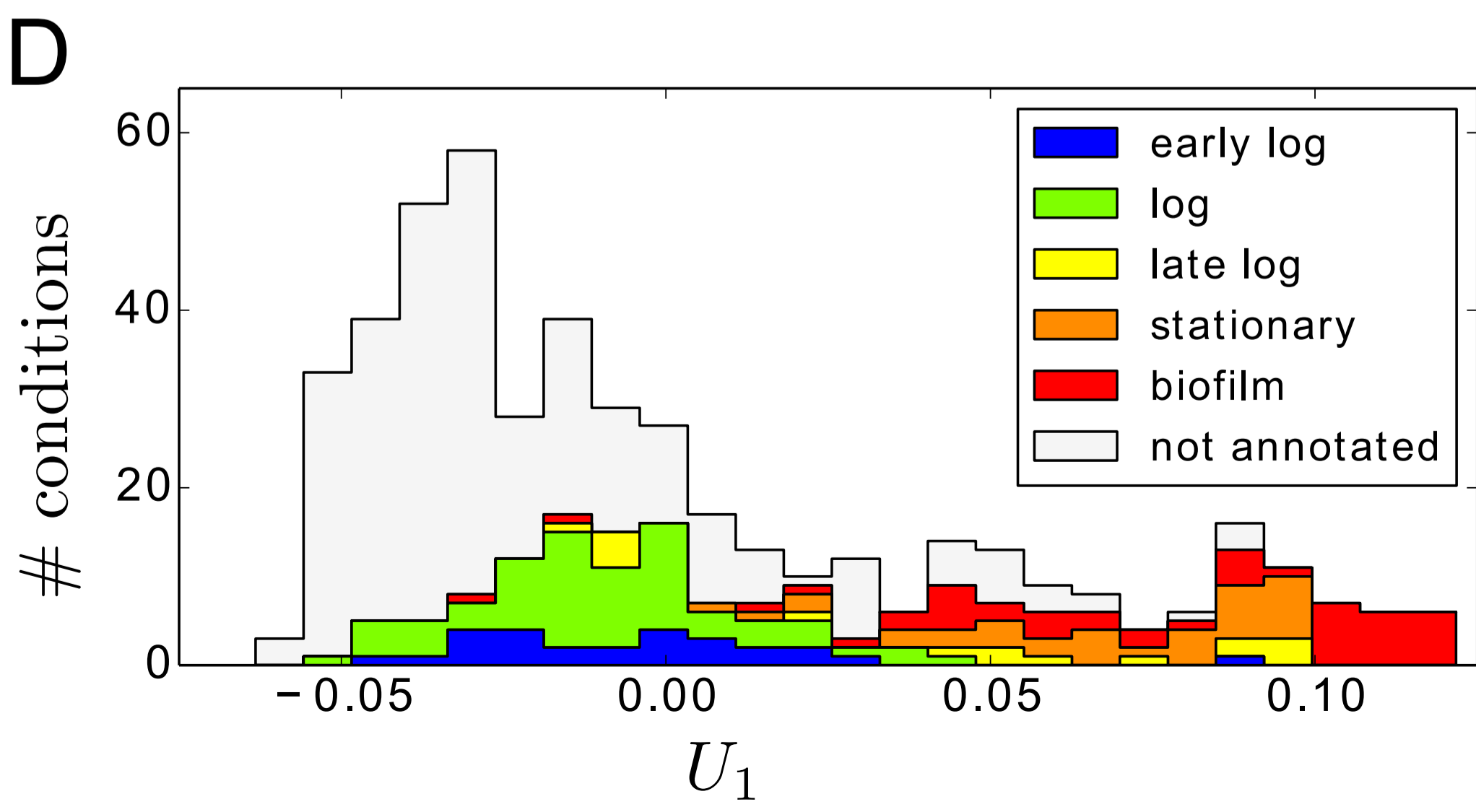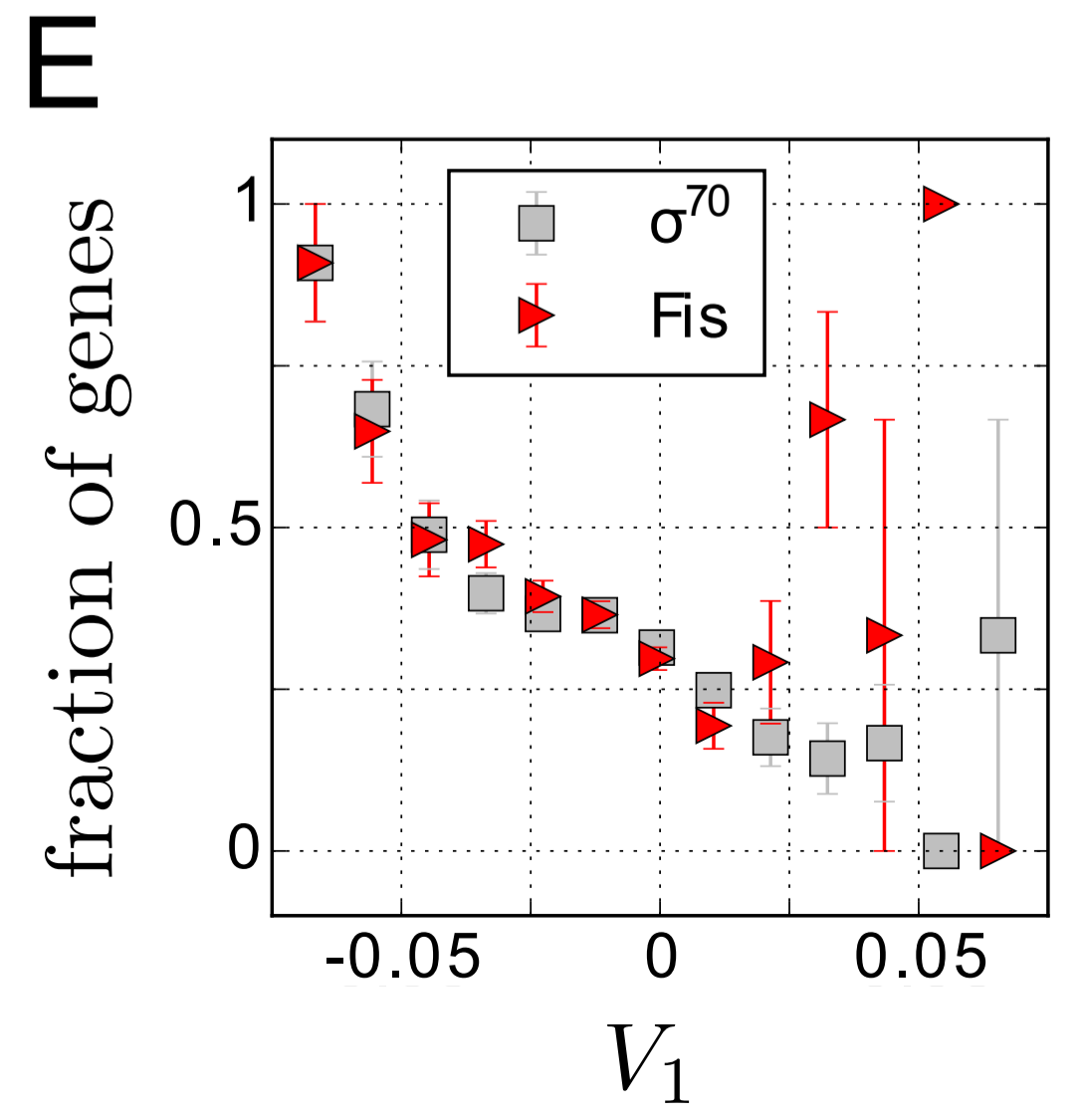

Supplement: S1 Fig — A. As in Fig 1A for E. coli, micro-array data reporting the expression levels of 4320 genes (rows) in 466 conditions (columns) with high expression in red and low expression in green. B. Applying a singular value decomposition to the micro-array data yields two principal components, V1 along the genes and U1 along the conditions. The co-expression matrix of Fig 1B is shown here with, above the diagonal, the genes sorted by V1: this component classifies the genes according to their contribution to one of the two anti-correlated clusters visible in Fig 1D. C. Same expression data as in A, but with the conditions sorted by U1 and the genes sorted by V1, thus revealing the main pattern of variation. D. Distribution of the conditions along the principal component U1, with different colors for the different phases of growth at which the measurements of transcriptional activity were made, showing that U1 correlates with the growth rate. E. Fraction of genes controlled by σ70 (gray squares) and with a binding site for the NAP Fis (red triangles) as a function of V1, showing that genes that are transcribed in growing phases (negative values of V1) are more likely to be regulated by σ70 and bound by Fis. (PDF) [file pone.0155740.s004.pdf]

# Fraction of gene pairs outside operons with conserved proximity

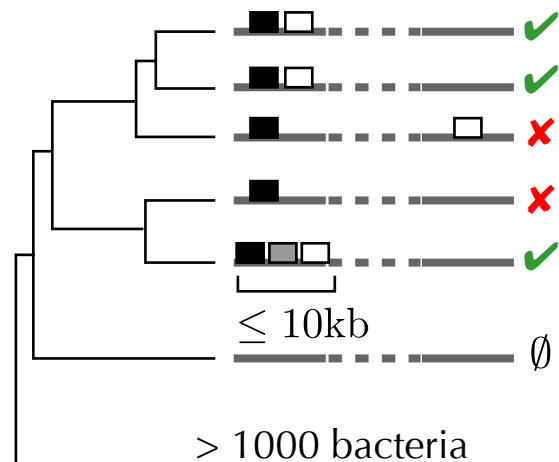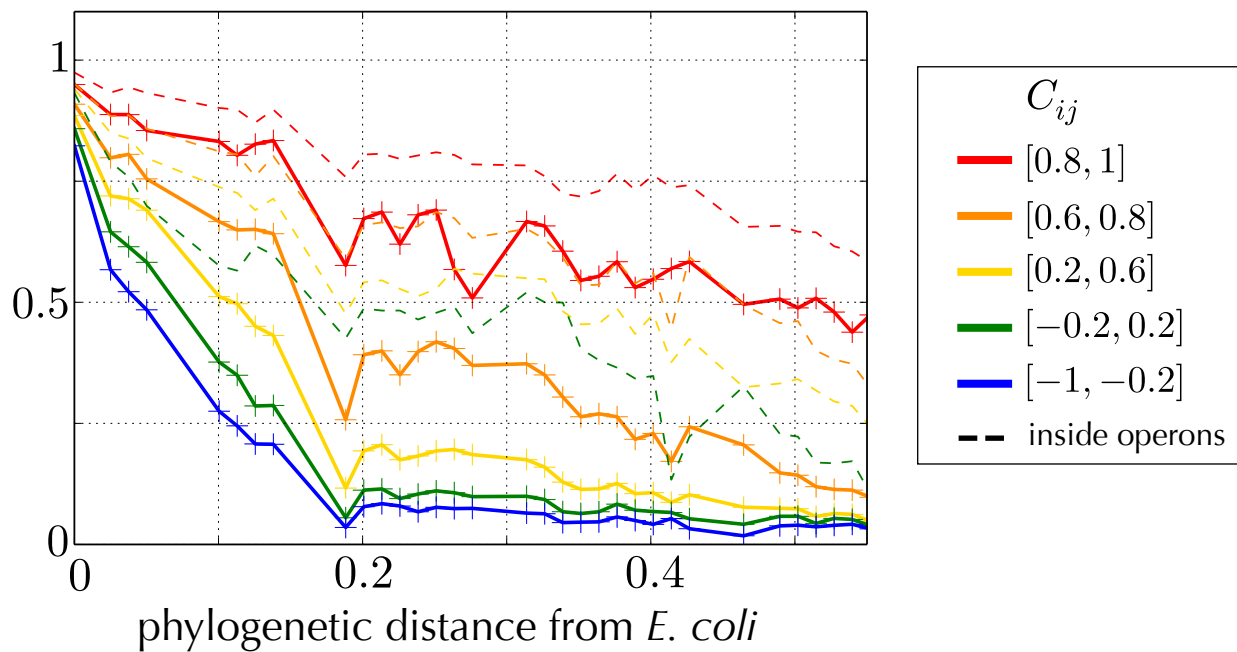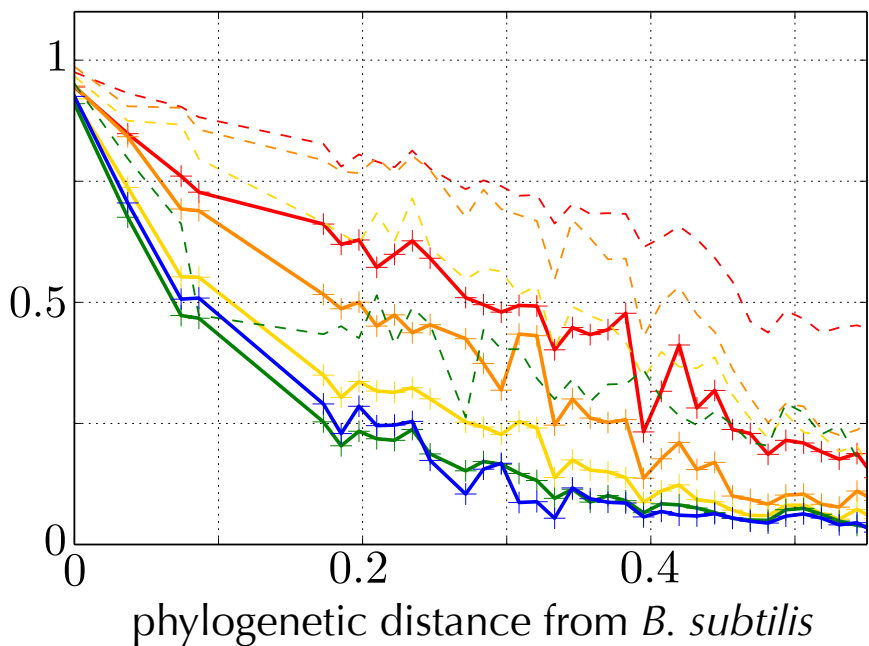

Supplement: S3 Fig — Synteny as a proxy for high co-expression. Taken two genes within 10 kb along the chromosome of a reference genome, what is the probability that these genes have orthologs within the same distance in the chromosome of another bacterium? We obtain an answer from a statistics over > 1000 bacterial genomes (left panel). This answer depends not only on the phylogenetic divergence between the query and reference genomes, but also very strongly on the level of co-expression of the two genes in the reference genome (plots): the more co-expressed are the two genes in E. coli (top) or in B. subtilis (bottom), the more likely they are to remain proximal in the chromosome of distant bacteria. The curves in the graph represent the fraction of pairs of genes within 10 kb in the reference genome (E. coli or B. subtilis) that are also within 10 kb in another genome as a function of the phylogenetic divergence between the two genomes (this divergence is measured by sequence divergence, see Materials and methods). Different colors correspond to pairs of genes with different levels of co-expression in the reference genome: proximity between highly co-expressed pairs, in red, is thus much more conserved than between weakly co-expressed pairs, in yellow. The plain lines are based on pairs of genes that do not belong to the same operon, and the dotted lines on pairs of operonic genes: this shows that the relation between co-expression and synteny extends beyond operons. (PDF) [file pone.0155740.s006.pdf]

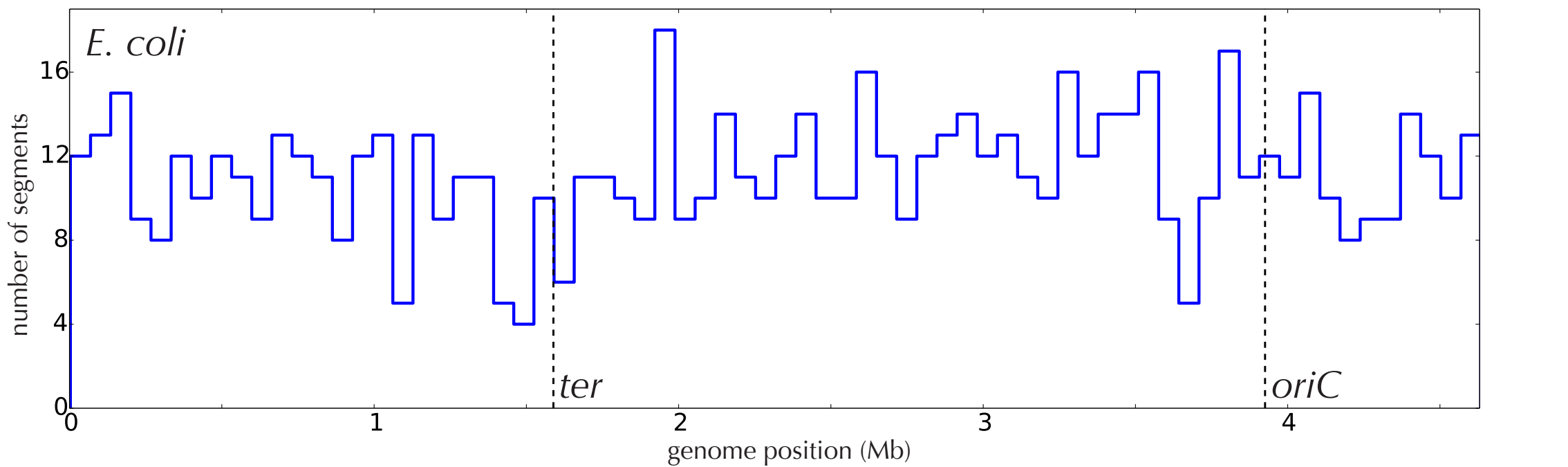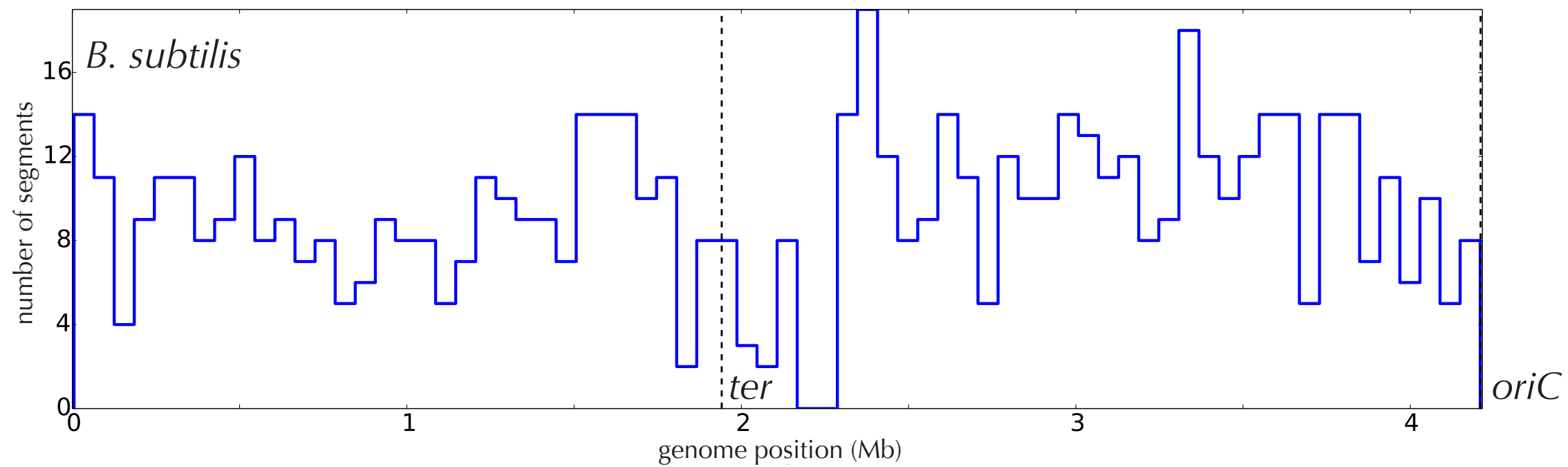

Supplement: S4 Fig — Genomic distribution of segments in E. coli (top) and in B. subtilis (bottom): the histograms of the location of the segments along the chromosome reveal a fairly uniform distribution (bin size of 65 kb). The vertical dashed lines indicate the origin (oriC) and terminus (ter) of replication. In B. subtilis, the depletion close to ter is mainly due to a poor gene annotation in this region. (PDF) [file pone.0155740.s007.pdf]

# Size of segments/operons

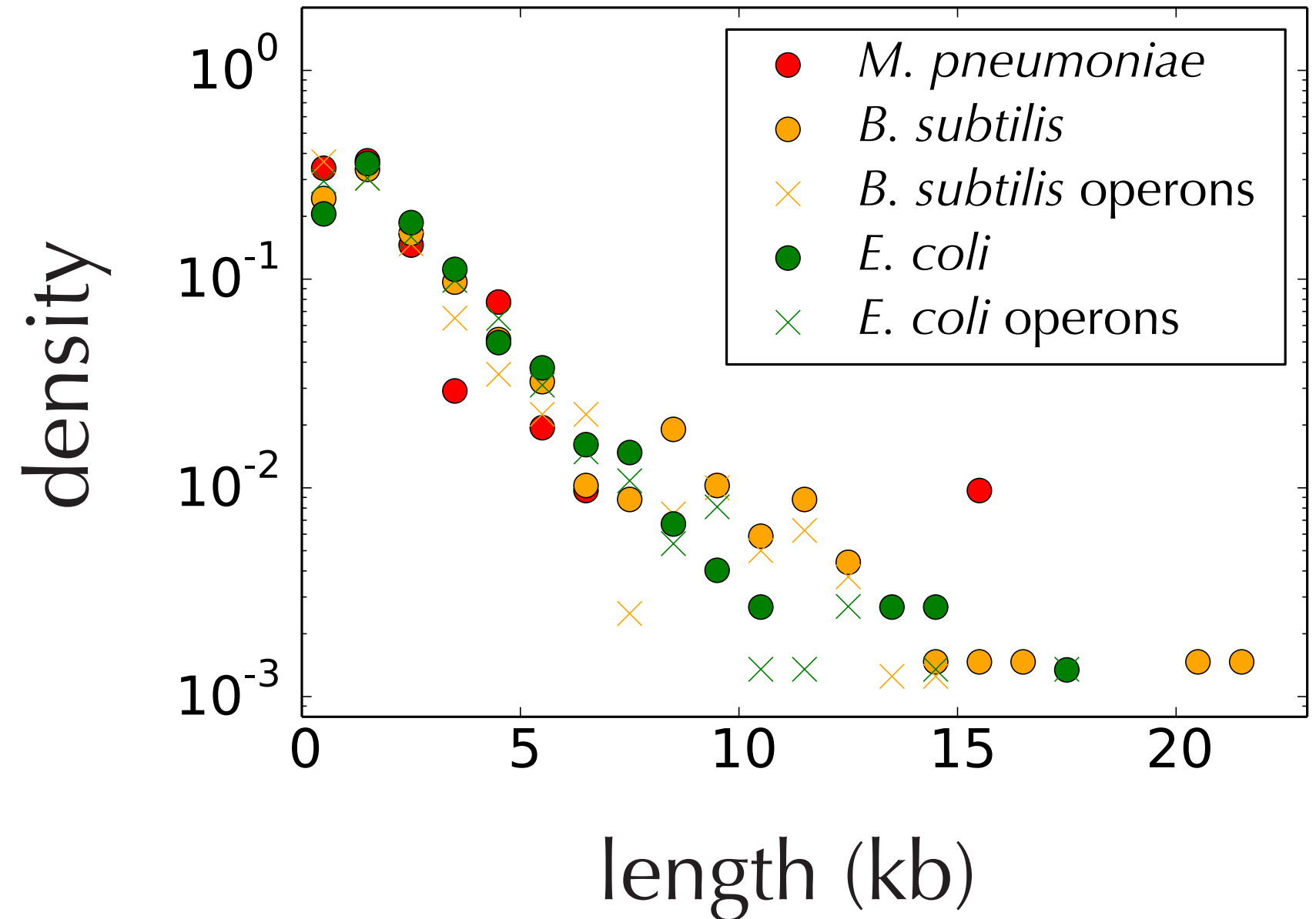

Supplement: S5 Fig — Size distributions of synteny segments (solid circles) in three phylogenetically distant bacteria and of polycistronic operons in E. coli and in B. subtilis (crosses), showing a similar exponential decrease up to ∼ 10 kb. (PDF) [file pone.0155740.s008.pdf]

# tsEPOD

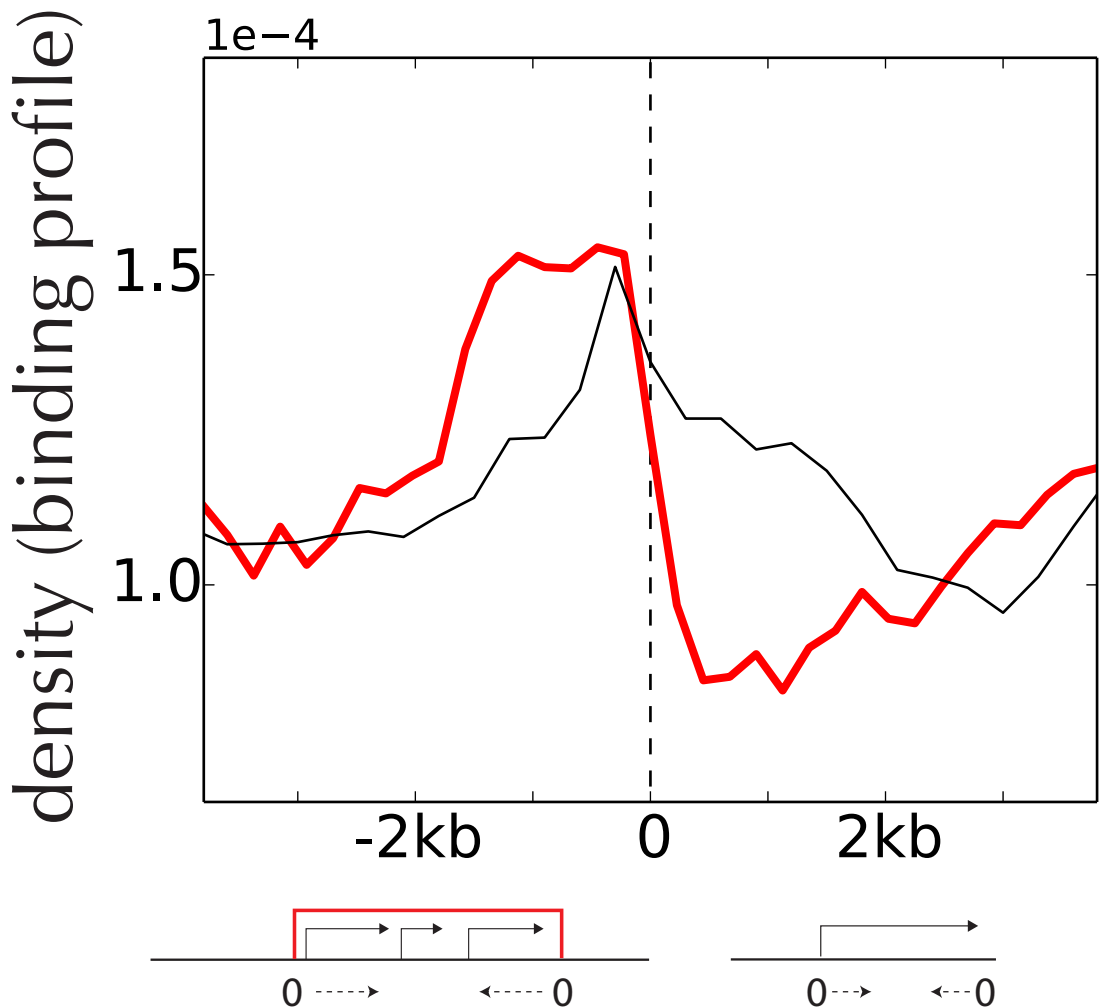

Supplement: S6 Fig — Binding profile of tsEPODs [53] with respect to synteny segments and operons, showing, as in the case of H-NS (Fig 2D), a strikingly high density of tsEPODs at the external boundaries of segments together with a depletion inside segments (in red). In agreement with their role in transcription silencing [94], we also observe an enrichment around the promoter region, and over the first gene for operons not at the border (in black). (PDF) [file pone.0155740.s009.pdf]

**A** *M. pneumoniae* (inter-operonic pairs)

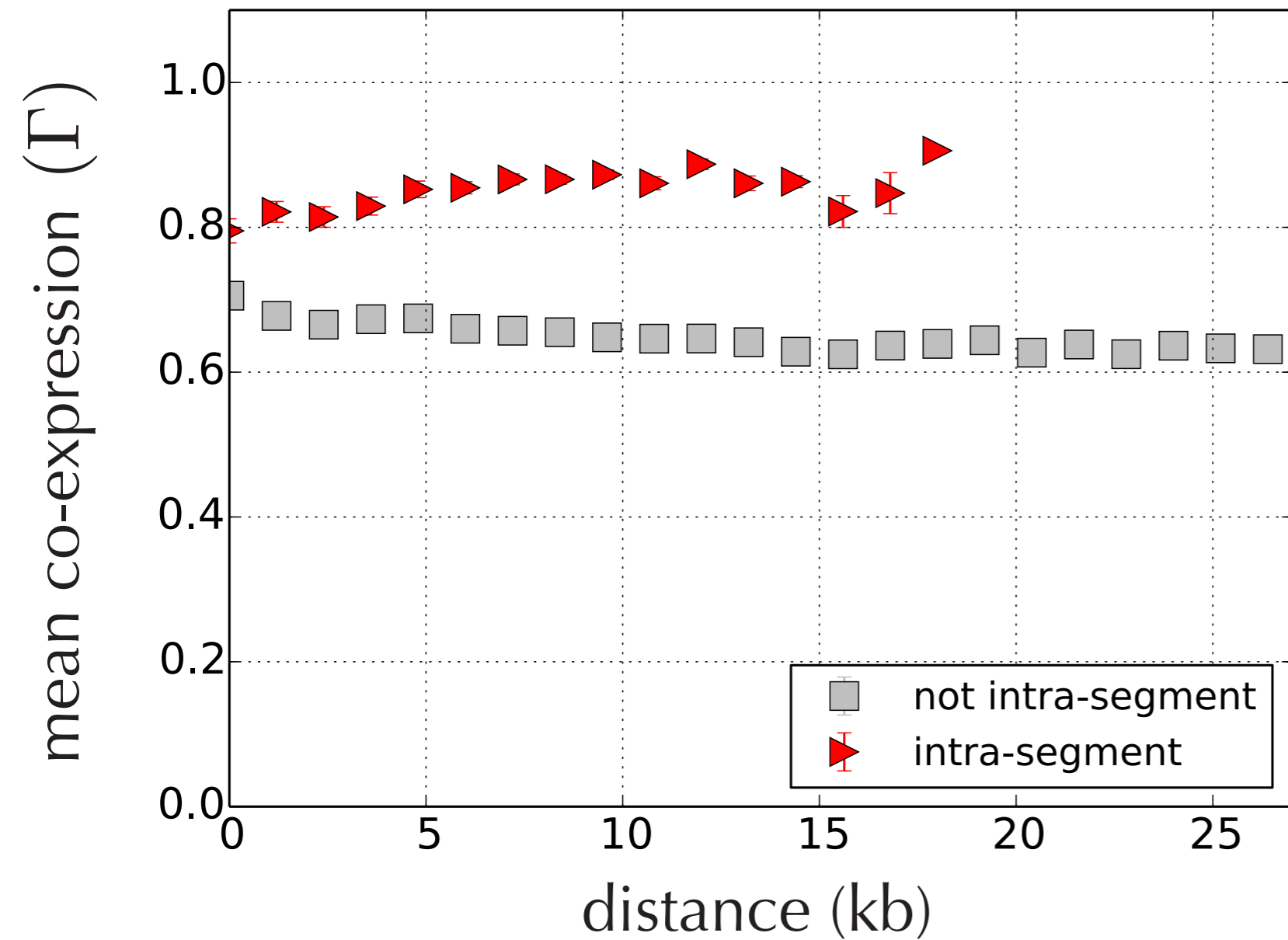

**B** *D. dadantii* (inter-operonic pairs)

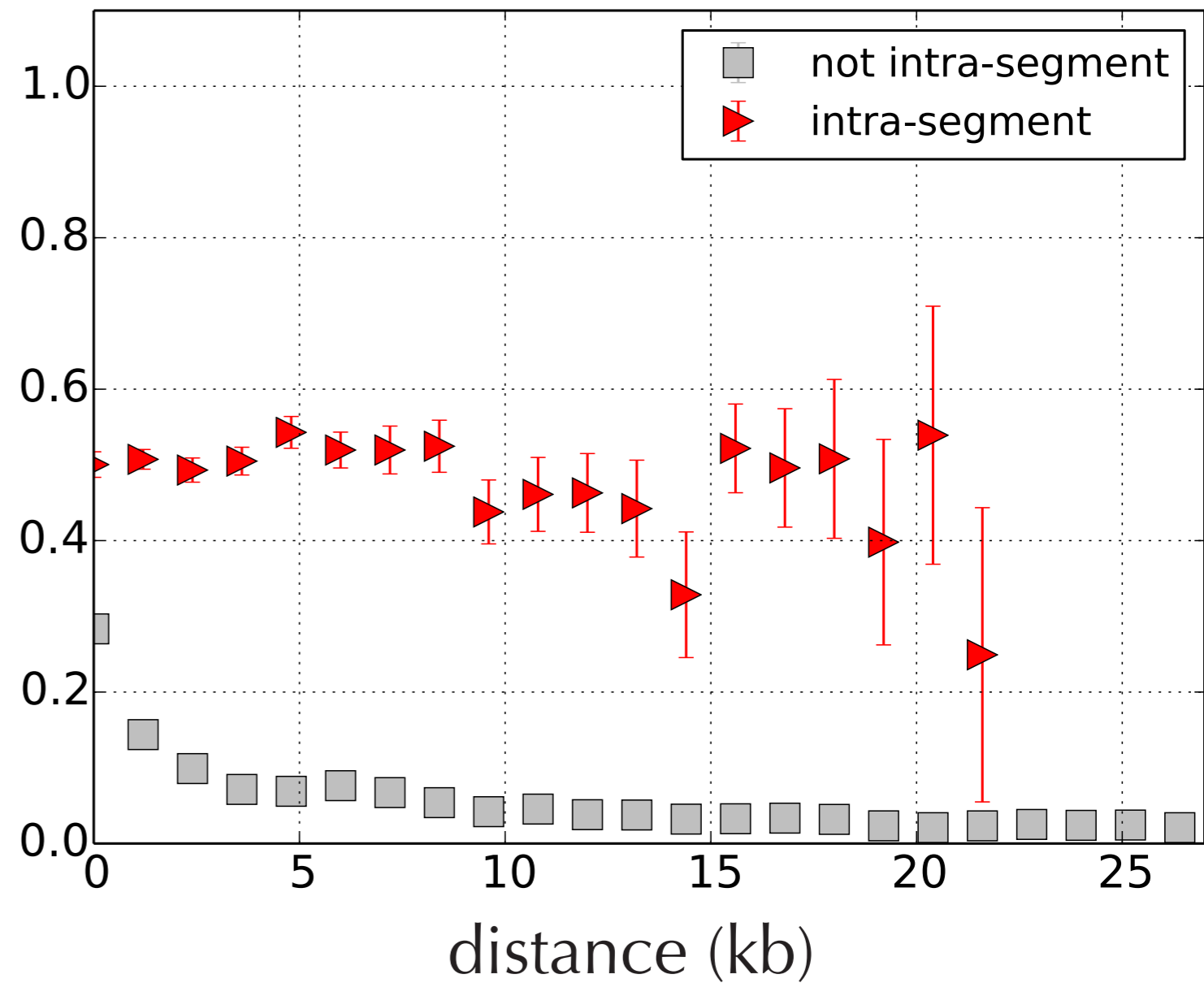

Supplement: S7 Fig — Co-expression analysis for two additional bacteria: A. Mycoplasma pneumoniae (classified as close to Gram-positive) and B. Dickeya dadantii (formerly Erwinia chrysanthemi, Gram-negative). These two bacterial strains have very different genome lengths (they contain respectively ca. 650 and 4500 protein coding genes) and lifestyles (M. pneumoniae is a human parasit living in the respiratory tract, D. dadantii is a plant pathogen); they are also phylogenetically distant from both E. coli and B. subtilis (analyzed in Fig 4). M. pneumoniae is known to have a tiny repertoire of TFs and a single major SF, while the regulatory network of D. dadantii is mostly unknown (as is the case for most bacteria). The graphs compare co-expression inside synteny segments (red triangles) to co-expression outside segments (gray squares). In both cases, only genes belonging to different operons are considered (operon map from [25] for M. pneumoniae and from the ProOpDB database [95] for D. dadantii). Co-expression levels are computed from rRNA normalized RNA-seq data obtained in 151 different conditions for M. pneumoniae [25] and from rRNA normalized micro-array data obtained in 32 different conditions for D. dadantii [96]. Although global levels of co-expression differ between strains (see [25] for a detailed analysis of co-expression properties in M. pneumoniae), a systematic enhancement of co-expression is observed inside synteny segments, which is nearly independent of the distance separating the genes. (PDF) [file pone.0155740.s010.pdf]

(inter-operonic co-expression)

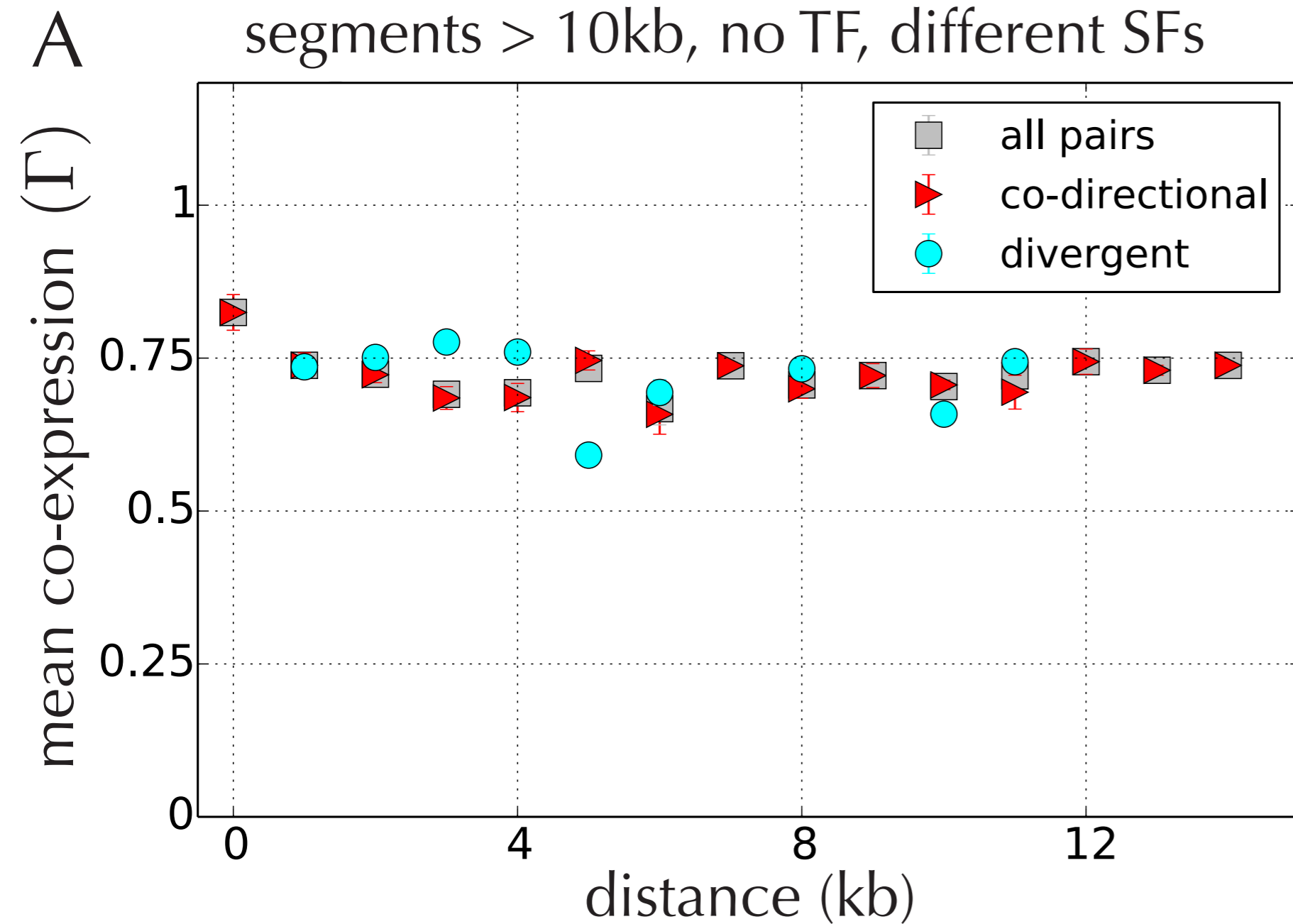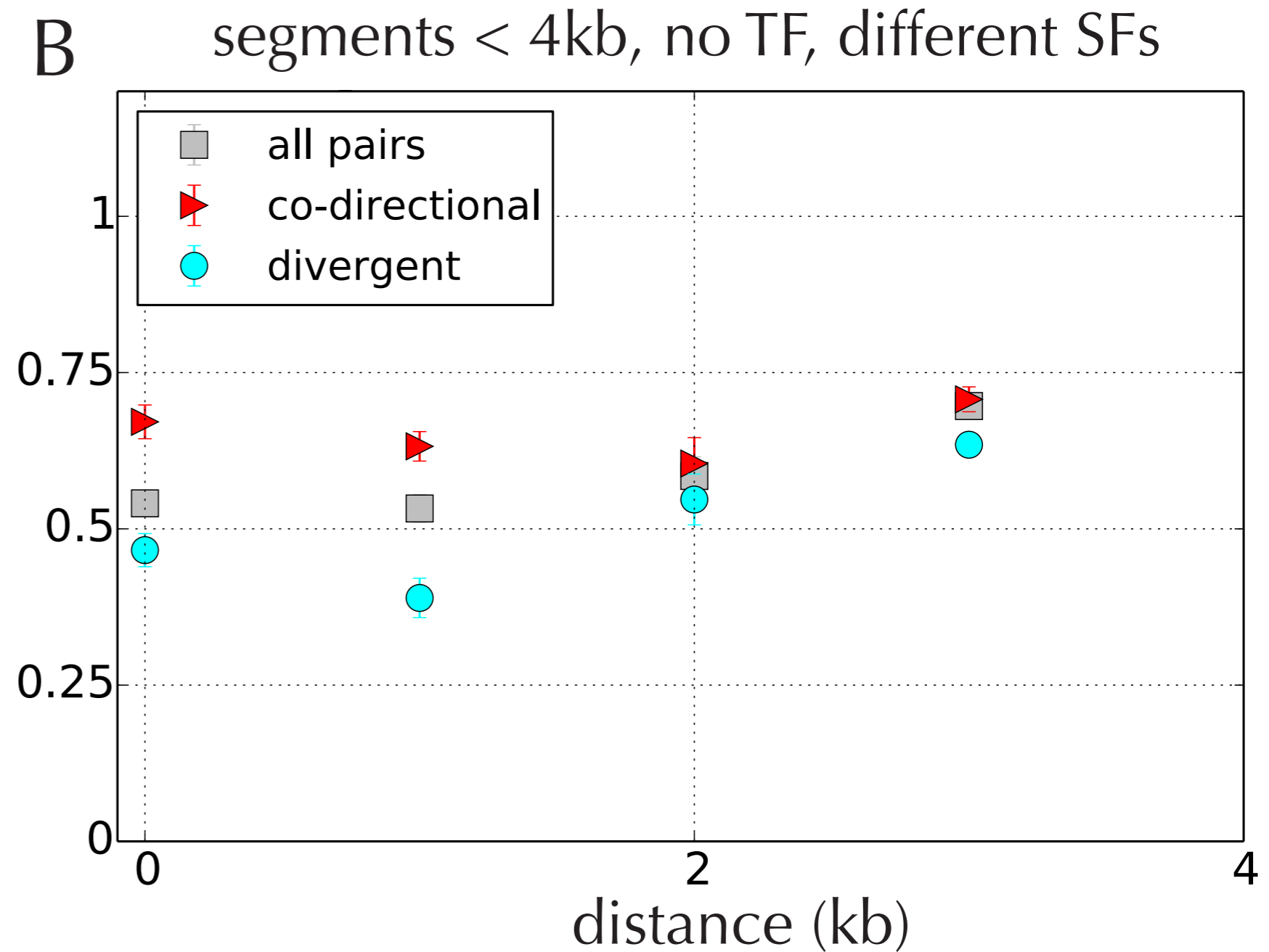

Supplement: S8 Fig — A. The red triangles correspond to those of Fig 4B (E. coli), and the gray squares and cyan points show that restricting to co-directional or divergent pairs has little incidence. B. Similar to A, but considering the smallest segments (< 4 kb) instead of the largest ones (> 10 kb): the overall level of correlation is lower for shorter segments. (PDF) [file pone.0155740.s011.pdf]

inter-operons, no TF, different SFs

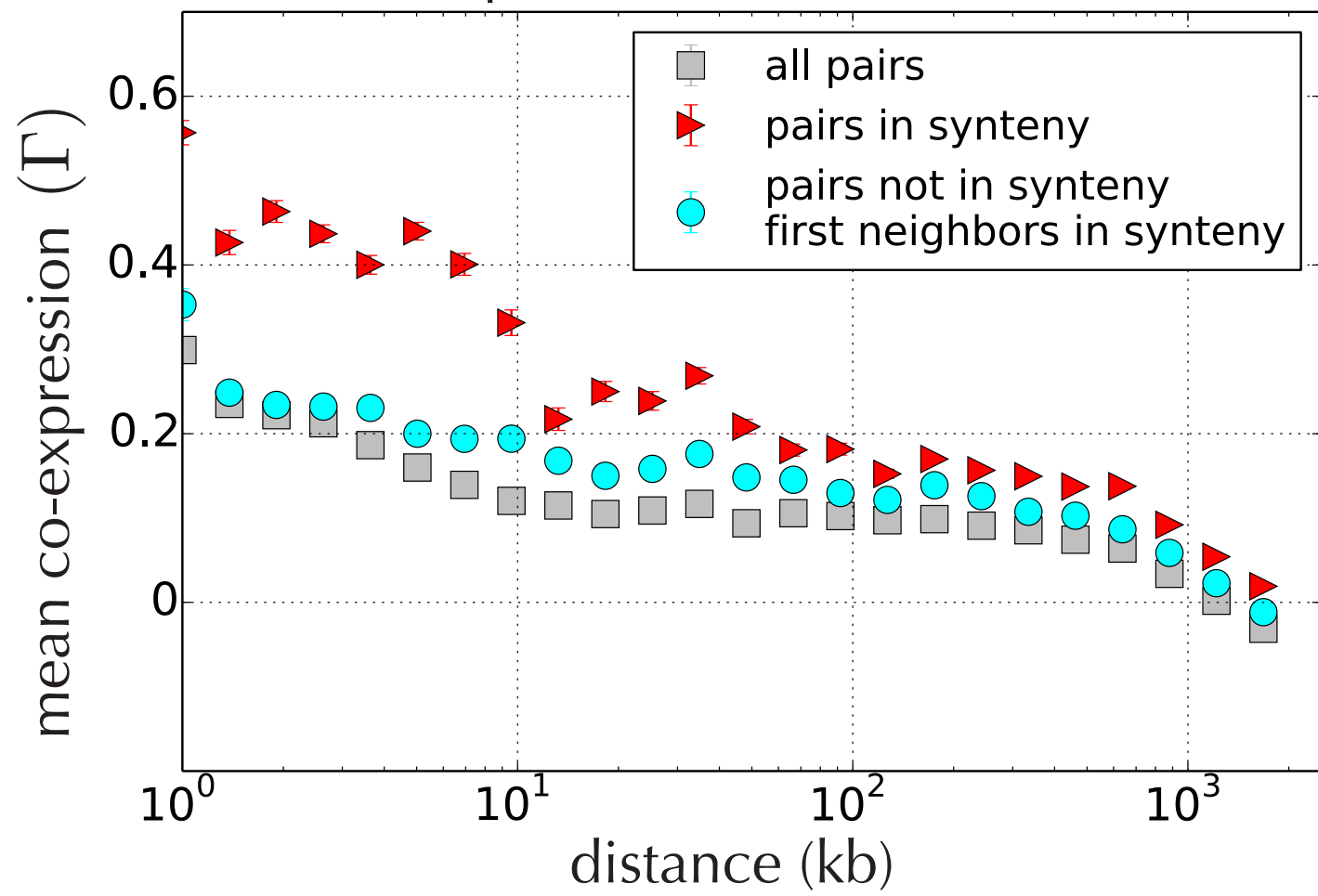

Supplement: S10 Fig — Co-expression between E. coli genes in different operons that are not regulated by any TF and that do not share the same SF (gray squares). Pairs in synteny, independently of whether they are proximal in the chromosome of E. coli, are on average more co-expressed than those not in synteny (red triangles). The phenomenon appears to be specific since replacing the first gene in these pairs by its nearest neighbor not in synteny (while keeping the second gene) systematically decreases the mean level of co-expression at all distances. (PDF) [file pone.0155740.s013.pdf]

fraction of adjacent genes in  $\neq$  operons

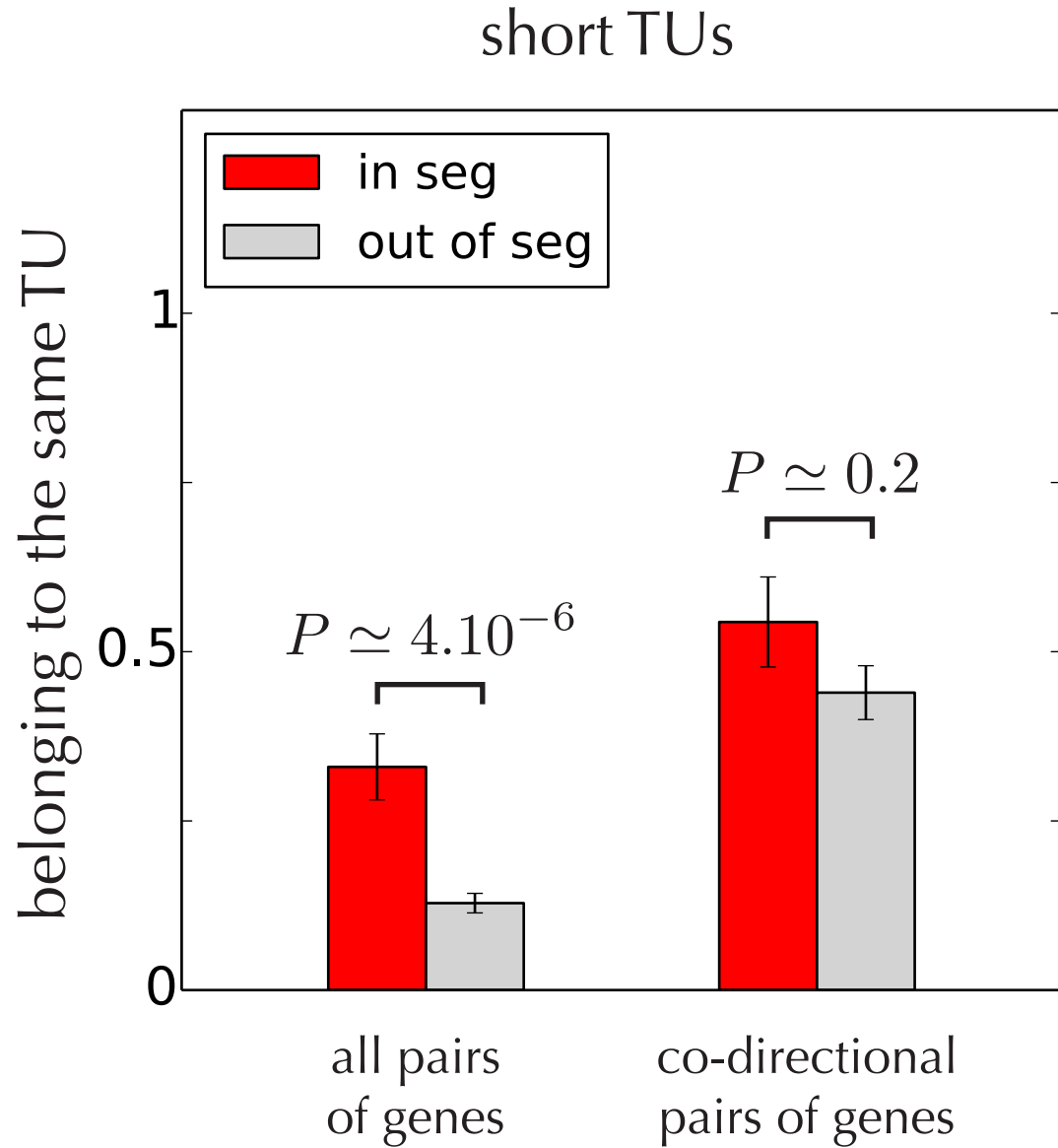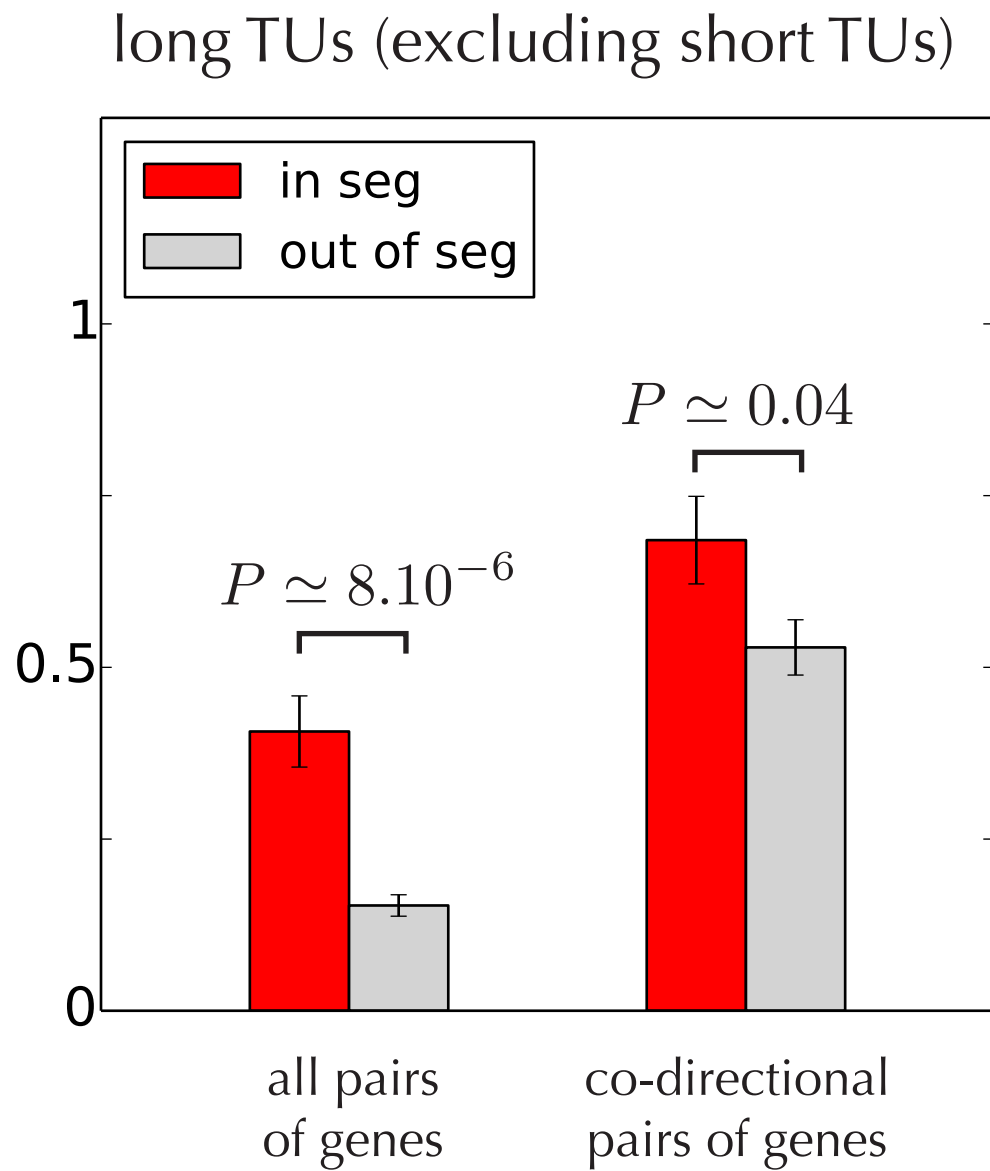

Supplement: S11 Fig — Fraction of adjacent genes that belong to a same transcriptional unit (TU) in B. subtilis [9]. Two types of TUs are considered as proposed in [9]: “short TUs” (left panel), which are minimal TUs found in most conditions, and “long TUs” (right panel), which are maximal TUs found in at least one condition. The fraction is computed for genes inside synteny segments (red bars) and for genes outside synteny segments (gray bars). In each panel, the two bars on the left are based on all pairs of genes in different operons and those on the right on pairs of co-directional genes in different operons. (PDF) [file pone.0155740.s014.pdf]

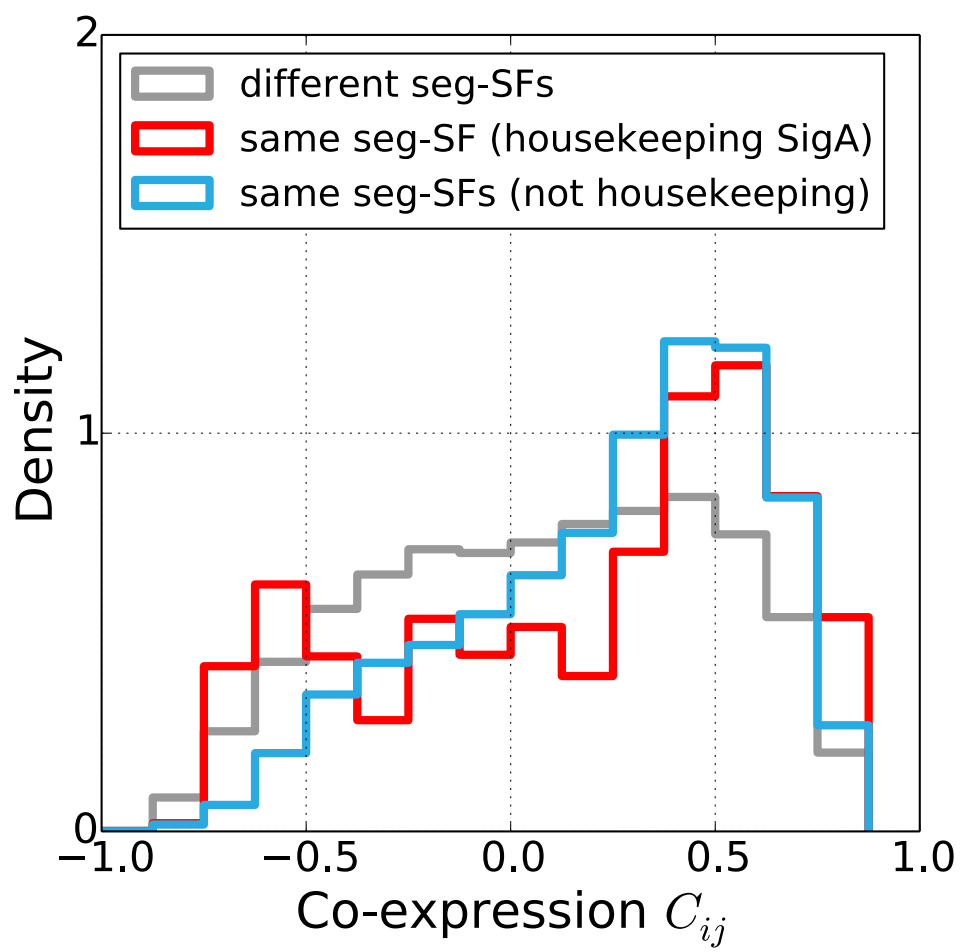

Supplement: S13 Fig — Distribution in B. subtilis of the co-expression Cij between pairs of genes that are not directly regulated by a TF or a SF and that belong to different synteny segments. Gray distribution: pairs in segments with different sets of SFs. Red distribution: pairs in segments that have one single seg-SF, the housekeeping SigA. Cyan distribution: pairs in segments that have exactly the same seg-SFs, excluding SigA. (PDF) [file pone.0155740.s016.pdf]

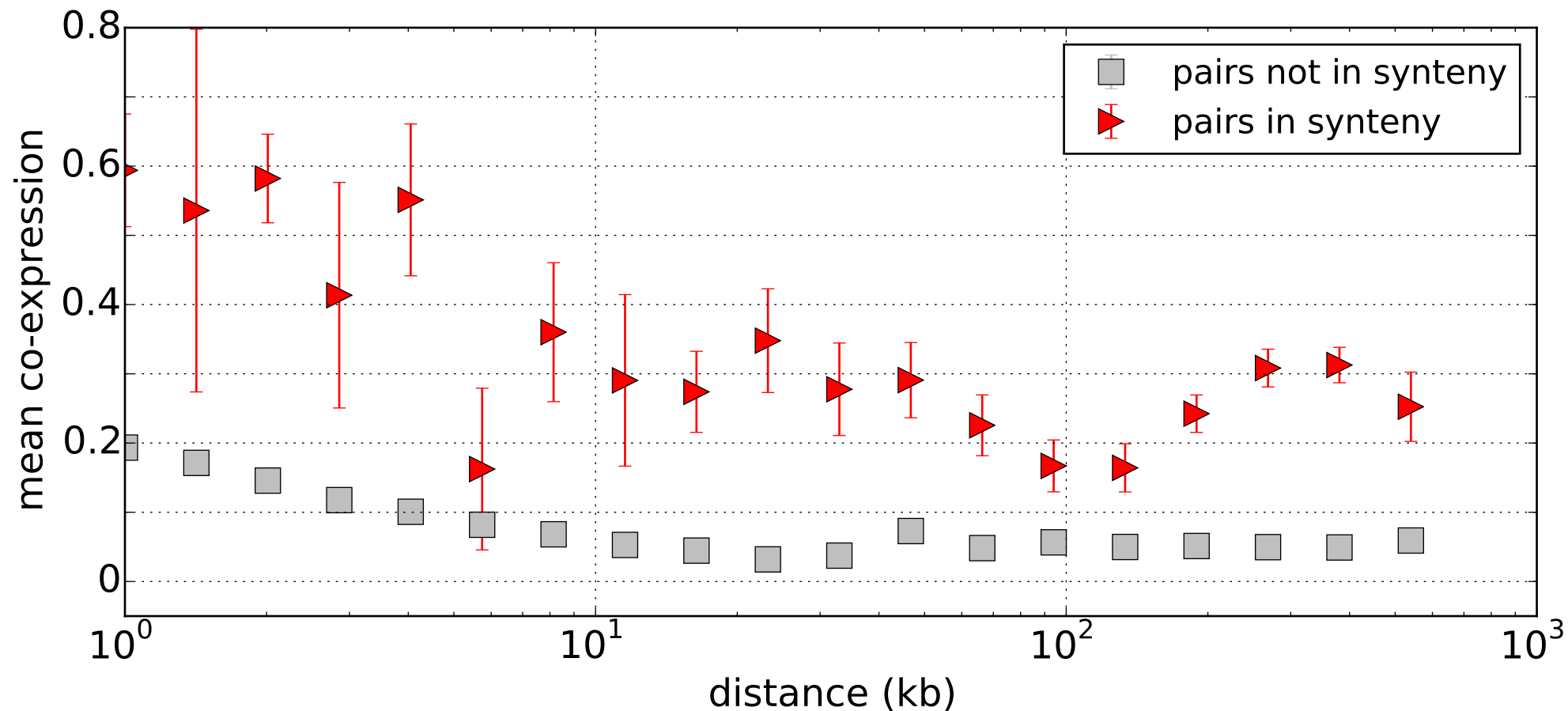

Supplement: S14 Fig — Co-expression for pairs of genes in synteny (red triangles) or not (gray squares) in S. cerevisiae. Synteny is defined from our dataset of bacterial genomes, which does not include any yeast genome. Co-expression is computed from micro-array data retrieved from the M3D database [8]. Pairs of genes in synteny in bacteria are in average more co-expressed in S. cerevisiae than pairs that are not in synteny in bacteria. (PDF) [file pone.0155740.s017.pdf]

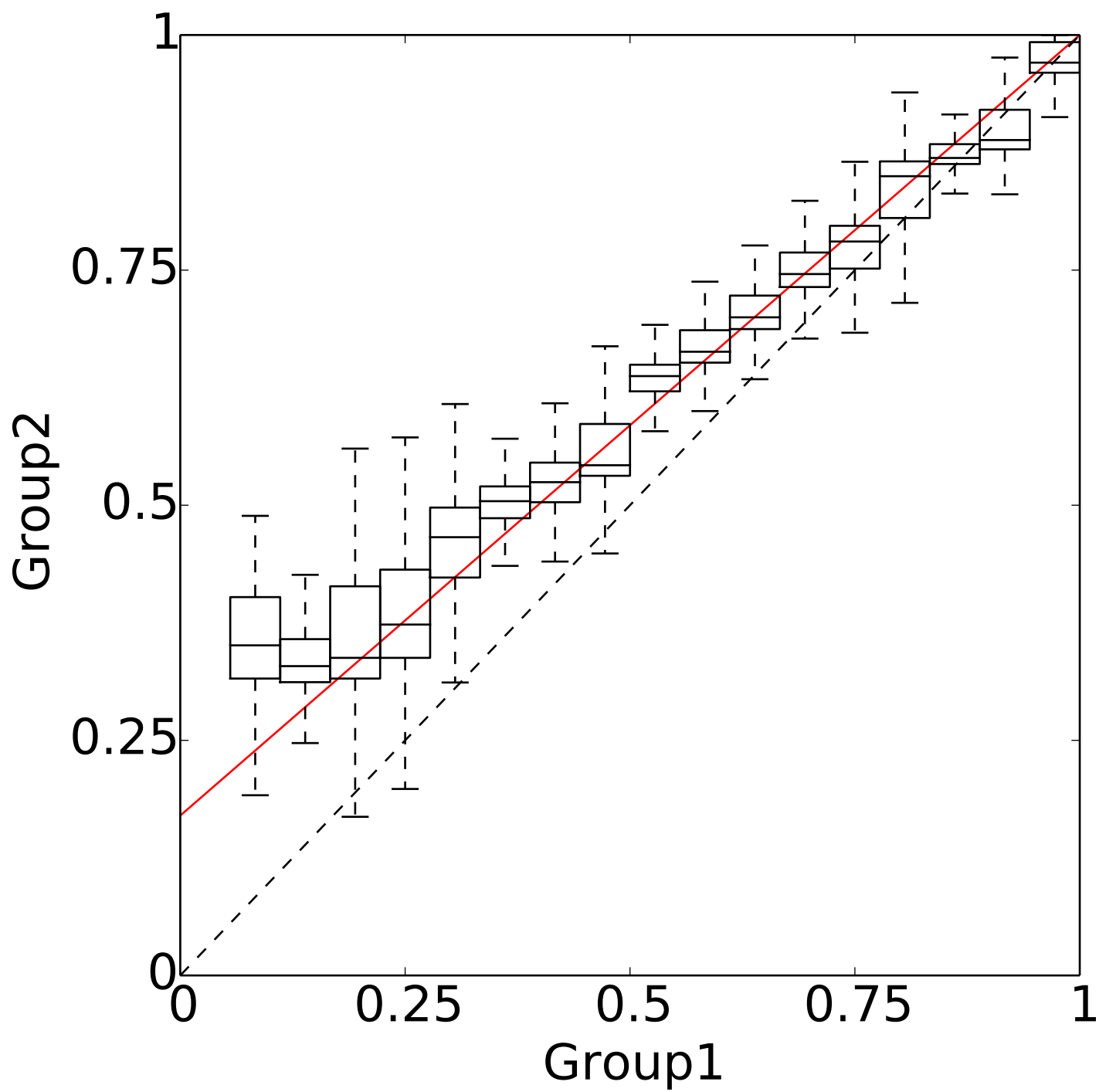

Supplement: S15 Fig — Robustness of the calculation of evolutionary distances. We compare two evolutionary distances that were computed using two disjoint groups of 5 genes that reflect phylogenetic distances between bacterial strains (Materials and methods). One can observe a linear relationship (in red) for almost the full range of similarities, except at very low similarities. All genome pairs formed from the 1445 genomes of our dataset are reported. The dashed black line indicates the bisector y = x. (PDF) [file pone.0155740.s018.pdf]

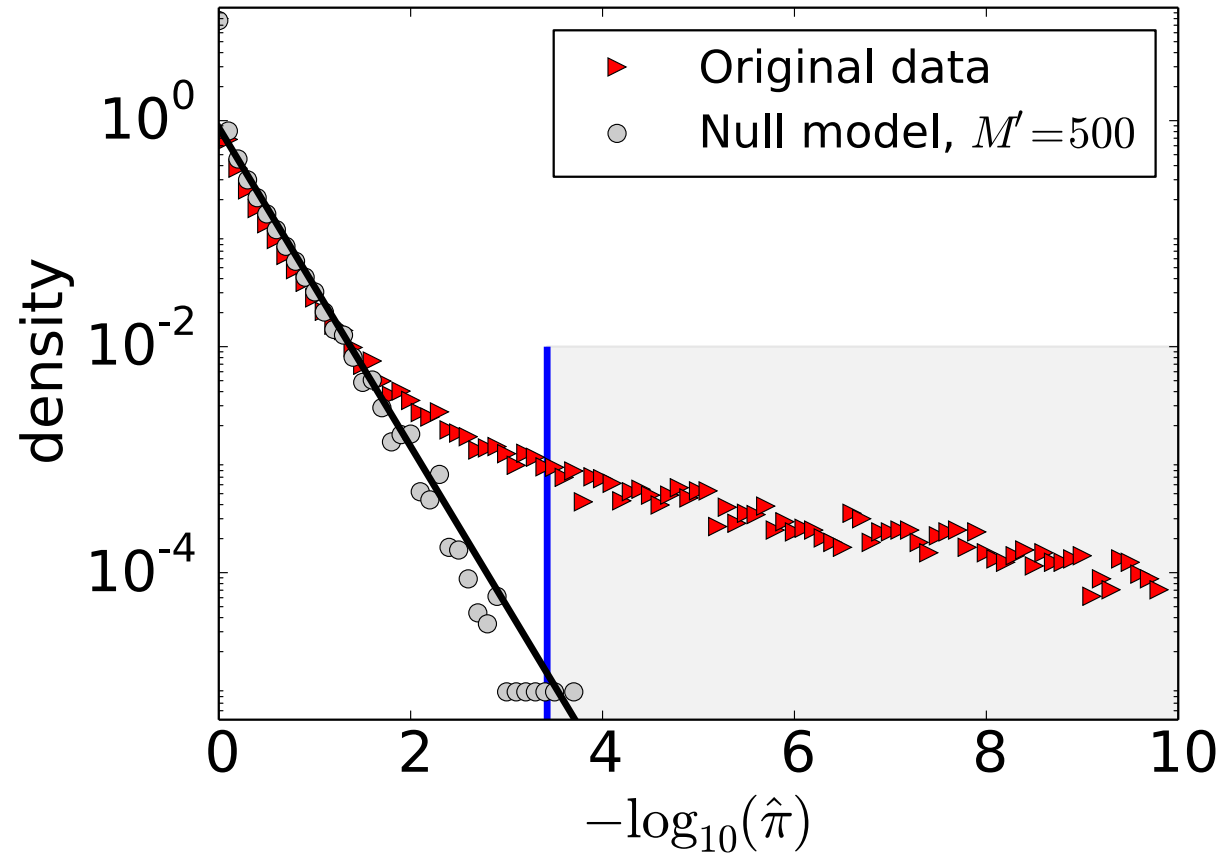

Supplement: S16 Fig — Probability density of -log(π^) for the empirical data (red triangles) obtained for an effective number of genomes M′ = 500. For small enough values of -log(π^), the density decays exponentially with -log(π^) (black line). The deviation from an exponential at large values (gray area) indicates the conservation of co-localization. For the null model (gray points), for which we consider the same effective number of genomes but where gene positions are randomized, the exponential decay extends to larger values of -log(π^). Here, we consider a false discovery rate FDR = 0.005, leading to a threshold π* ≃ 4.10−4 (vertical blue line). (PDF) [file pone.0155740.s019.pdf]
