# Supplementary material for: Conserved Units of Co-Expression in Bacterial Genomes: An Evolutionary Insight into Transcriptional Regulation
Source: PLoS One. 2016 May 19;11(5):e0155740. doi: 10.1371/journal.pone.0155740 (PMC4873041; doi:10.1371/journal.pone.0155740)
Supplement: S2 Fig — A. Transcriptional co-expression between the 1231 genes of E. coli having σ70 as unique SF. Genes are reordered along the first component V1 from the SVD decomposition of the data as in S1B Fig. B. In E. coli, fraction of pairs of genes belonging to different operons that share a TF, a SF or one of the two, showing that, except at very high level of co-expression (Cij > 0.85), the majority (∼ 75%) of correlated pairs of genes do not share a common TF or SF. C. Same analysis in B. subtilis. (PDF) [file pone.0155740.s005.pdf]

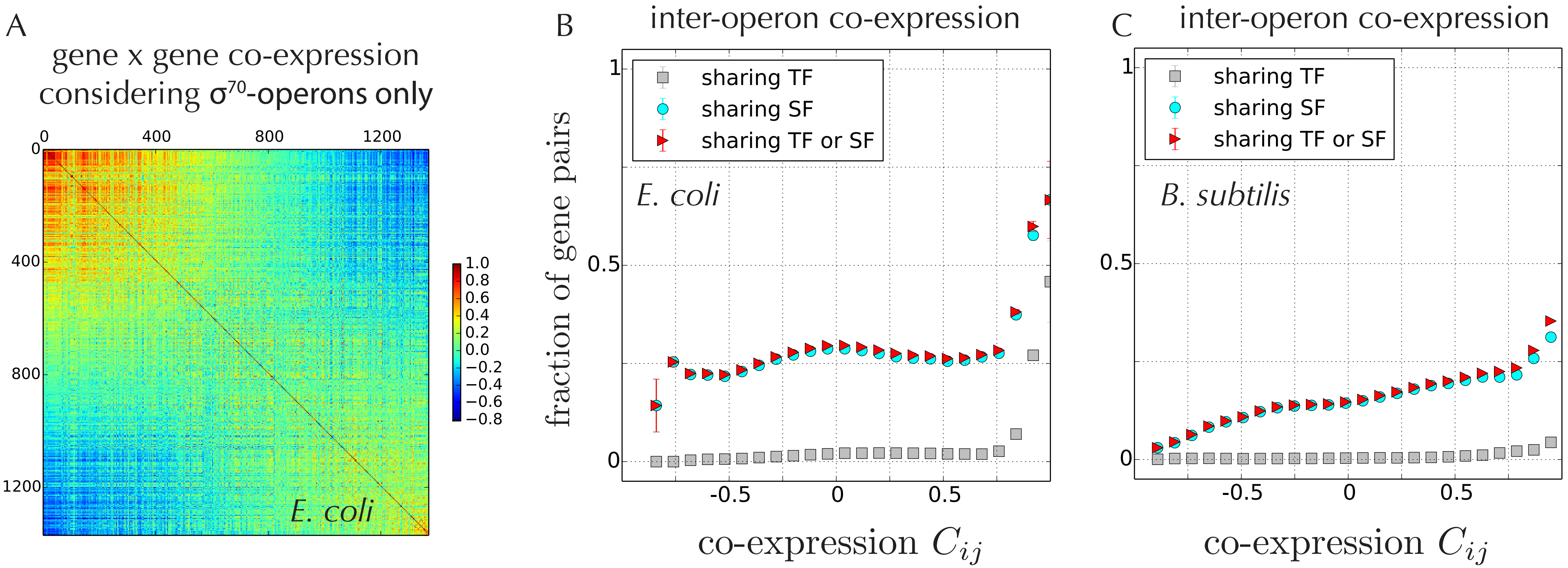

A
gene x gene co-expression
considering σ70-operons only
0
400
800
1200
0
400
800
1200
1.0
0.8
0.6
0.4
0.2
0.0
−0.2
−0.4
−0.6
−0.8
E. coli
B
inter-operon co-expression
sharing TF
sharing SF
sharing TF or SF
E. coli
fraction of gene pairs
1
0.5
0
−0.5
0
0.5
co-expression C_{ij}
C
inter-operon co-expression
sharing TF
sharing SF
sharing TF or SF
B. subtilis
1
0.5
0
−0.5
0
0.5
co-expression C_{ij}
