# Supplementary material for: Conserved Units of Co-Expression in Bacterial Genomes: An Evolutionary Insight into Transcriptional Regulation
Source: PLoS One. 2016 May 19;11(5):e0155740. doi: 10.1371/journal.pone.0155740 (PMC4873041; doi:10.1371/journal.pone.0155740)
Supplement: S9 Fig — Average number of operons directly controlled by at least one TF (upper panels) or by at least one SF (lower panels) as a function of the number of operons in the segment. Results show that both in E. coli (left panels) and in B. subtilis (right panels) there is roughly a constant number (close to 1) of operons directly regulated by a TF. In contrast, most operons are directly regulated by a SF in E. coli (left lower panel). In B. subtilis, not all operons of the segment are regulated by a SF, but at least one. The dashed lines in the lower panels indicate the bisectors y = x. (PDF) [file pone.0155740.s012.pdf]

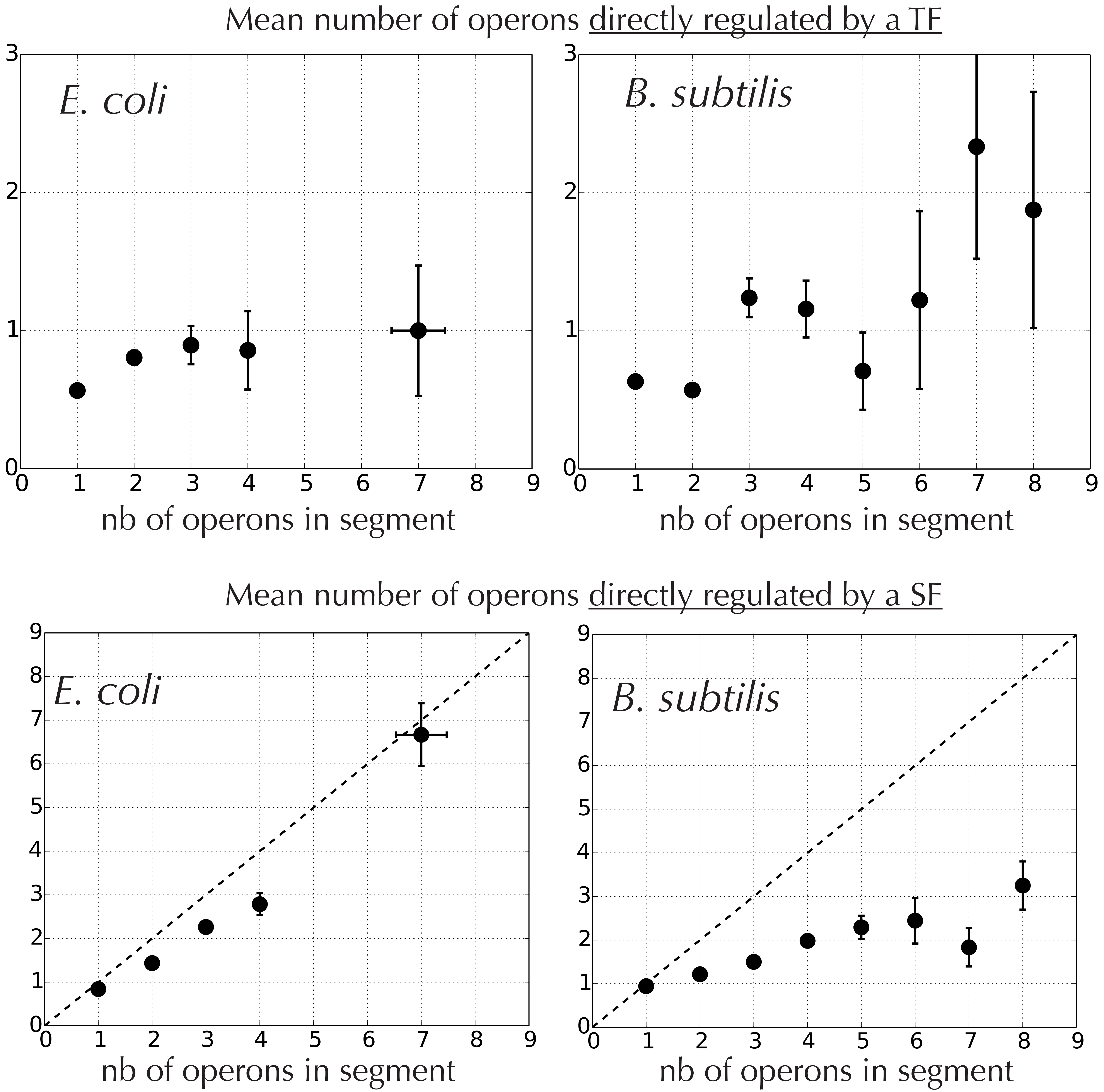
Mean number of operons directly regulated by a TF
E. coli
B. subtilis
nb of operons in segment
nb of operons in segment
Mean number of operons directly regulated by a SF
E. coli
B. subtilis
nb of operons in segment
nb of operons in segment
