# Supplementary material for: Conserved Units of Co-Expression in Bacterial Genomes: An Evolutionary Insight into Transcriptional Regulation
Source: PLoS One. 2016 May 19;11(5):e0155740. doi: 10.1371/journal.pone.0155740 (PMC4873041; doi:10.1371/journal.pone.0155740)
Supplement: S12 Fig — A. Extension of the results of Fig 6C, showing that conserved high co-expression is mostly due to a seg-regulation by housekeeping SFs (σ70 in E. coli and SigA in B. subtilis). B. Contribution of the seg-regulation by housekeeping SFs in each organism. C. Same as in B but considering only genes that belong to synteny segments, showing a strong relationship in both bacteria between gene co-expression and seg-regulation by a housekeeping SF. In B and C, the drop at high co-expression level for B. subtilis may either come from a too partial annotation of SF binding sites, or from the imperfect match between our synteny segments and the actual relevant co-expression unit of B. subtilis. (PDF) [file pone.0155740.s015.pdf]

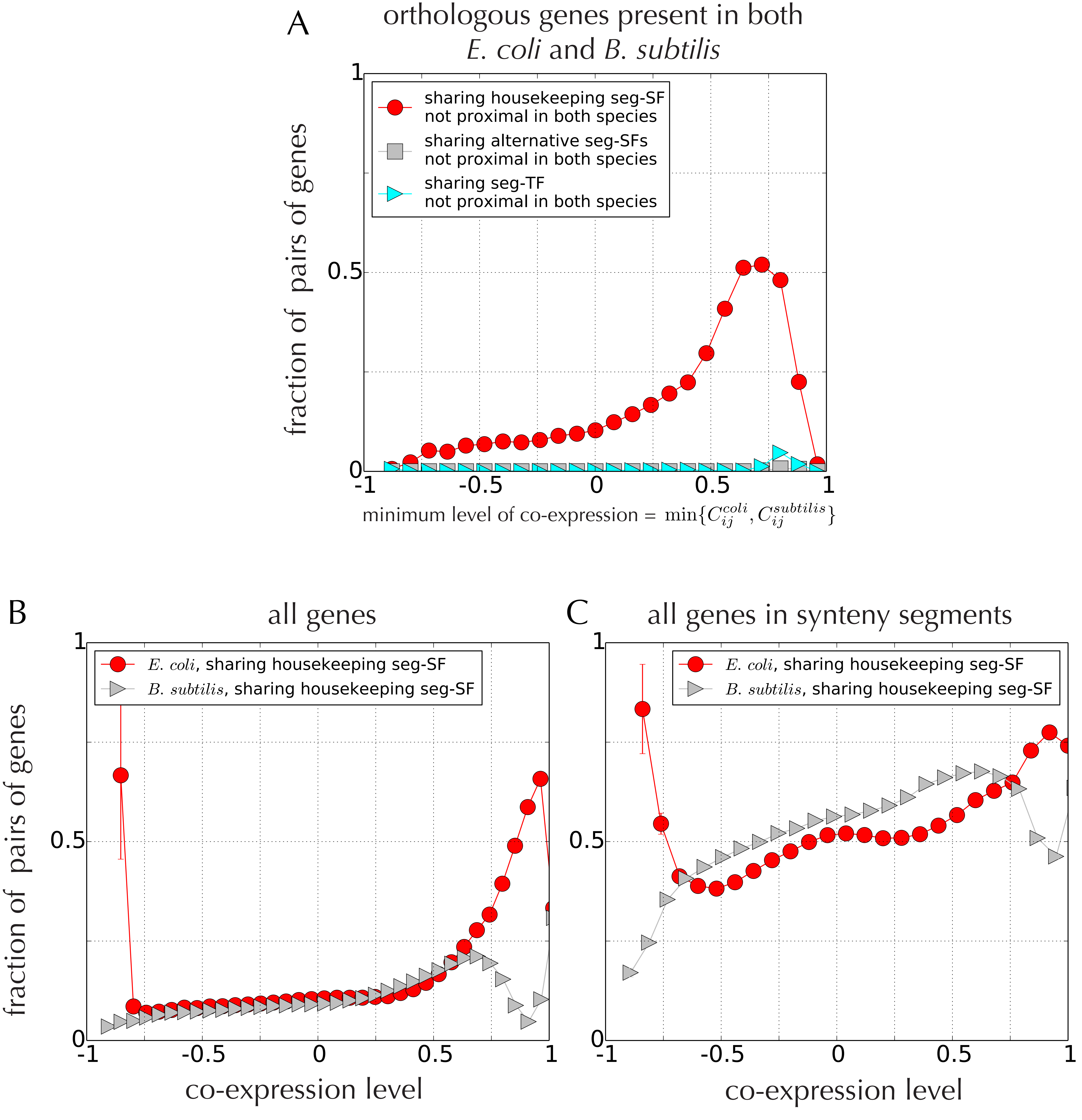
A
orthologous genes present in both
E. coli and B. subtilis
sharing housekeeping seg-SF
not proximal in both species
sharing alternative seg-SFs
not proximal in both species
sharing seg-TF
not proximal in both species
fraction of pairs of genes
minimum level of co-expression = $\min\{C_{ij}^{coli}, C_{ij}^{subtilis}\}$
B
all genes
E. coli, sharing housekeeping seg-SF
B. subtilis, sharing housekeeping seg-SF
fraction of pairs of genes
co-expression level
C
all genes in synteny segments
E. coli, sharing housekeeping seg-SF
B. subtilis, sharing housekeeping seg-SF
co-expression level
